# Grouping MWCNTs based on their similar potential to cause pulmonary hazard after inhalation: a case-study

**DOI:** 10.1186/s12989-022-00487-6

**Published:** 2022-07-20

**Authors:** Fiona Murphy, Nicklas Raun Jacobsen, Emilio Di Ianni, Helinor Johnston, Hedwig Braakhuis, Willie Peijnenburg, Agnes Oomen, Teresa Fernandes, Vicki Stone

**Affiliations:** 1 NanoSafety Group, Heriot-Watt University, Edinburgh, UK; 2 National Research Centre for the Working Environment (NFA), Copenhagen, Denmark; 3 National Institute for Public Health and the Environment (RIVM), Bilthoven, The Netherlands; 4 Institute of Environmental Sciences, Leiden University, Leiden, The Netherlands

**Keywords:** Grouping, Read-across, High aspect ratio nanomaterials

## Abstract

**Background:**

The EU-project GRACIOUS developed an Integrated Approach to Testing and Assessment (IATA) to support grouping high aspect ratio nanomaterials (HARNs) presenting a similar inhalation hazard. Application of grouping reduces the need to assess toxicity on a case-by-case basis and supports read-across of hazard data from substances that have the data required for risk assessment (source) to those that lack such data (target). The HARN IATA, based on the fibre paradigm for pathogenic fibres, facilitates structured data gathering to propose groups of similar HARN and to support read-across by prompting users to address relevant questions regarding HARN morphology, biopersistence and inflammatory potential. The IATA is structured in tiers, allowing grouping decisions to be made using simple in vitro or in silico methods in Tier1 progressing to in vivo approaches at the highest Tier3. Here we present a case-study testing the applicability of GRACIOUS IATA to form an evidence-based group of multiwalled carbon nanotubes (MWCNT) posing a similar predicted fibre-hazard, to support read-across and reduce the burden of toxicity testing.

**Results:**

The case-study uses data on 15 different MWCNT, obtained from the published literature. By following the IATA, a group of 2 MWCNT was identified (NRCWE006 and NM-401) based on a high degree of similarity. A pairwise similarity assessment was subsequently conducted between the grouped MWCNT to evaluate the potential to conduct read-across and fill data gaps required for regulatory hazard assessment. The similarity assessment, based on expert judgement of Tier 1 assay results, predicts both MWCNT are likely to cause a similar acute in vivo hazard. This result supports the possibility for read-across of sub-chronic and chronic hazard endpoint data for lung fibrosis and carcinogenicity between the 2 grouped MWCNT. The implications of accepting the similarity assessment based on expert judgement of the MWCNT group are considered to stimulate future discussion on the level of similarity between group members considered sufficient to allow regulatory acceptance of a read-across argument.

**Conclusion:**

This proof-of-concept case-study demonstrates how a grouping hypothesis and IATA may be used to support a nuanced and evidence-based grouping of ‘similar’ MWCNT and the subsequent interpolation of data between group members to streamline the hazard assessment process.

**Supplementary Information:**

The online version contains supplementary material available at 10.1186/s12989-022-00487-6.

## Background

The GRACIOUS Framework is a tool to facilitate the grouping of similar nanoforms (NFs) to support read-across and thereby streamline the hazard and risk assessment of existing and novel nanomaterials (NMs) [1]. More specifically, the formation of a group requires assessment of sufficient similarity between group members and will support the interpolation of data for regulatory hazard endpoints between group members for which data is available to group members where data is lacking [2]. The GRACIOUS Framework has a number of pre-defined grouping hypotheses which will support the user in making a grouping decision appropriate for their purpose and context. Tailored to a specific hazard endpoint of interest, the pre-defined grouping hypotheses support the grouping of NFs that operate via the same mode of action and share a common fate/hazard potential [1]. Exclusion of a NF from a group does not mean that it is not hazardous, but rather it’s hazard potential is not sufficiently similar to, or driven by the same mechanism of toxicity as, other NFs which align with the grouping hypothesis.

Multiwalled carbon nanotubes (MWCNT) represent the largest class of high aspect ratio nanomaterials (HARNs) currently in production, with the global market predicted to be worth over 16 billion USD by 2027, driven largely by demand in the automotive, textiles and electronics industries (https://www.emergenresearch.com/industry-report/carbon-nanotube-market). There is a huge diversity of MWCNTs available encompassing varied physicochemical properties such as fibre morphology (short, entangled, long, needle-like, differing number of graphene layers) and surface functionalities. The GRACIOUS Framework can be used to support the hypothesis-driven selective grouping of MWCNT predicted to pose a similar hazard following inhalation. This will result in the more streamlined and refined assessment of this large class of NMs which includes a variety of hazard profiles [3].

Potential aerosolization of MWCNT indicates occupational inhalation exposure to MWCNT is possible during primary production and the incorporation of MWCNT into nano-enabled products. Use in the occupational context suggests the high likelihood of repeated exposure for workers to potentially hazardous MWCNT. Therefore, a regulatory hazard endpoint of primary interest to MWCNTs is the *specific target organ toxicity after repeated exposure* (STOT-RE) with inhalation as the primary exposure route of concern (ECHA, 2016). OECD TG 412/413 are designed to assess repeat-dose toxicity of a substance by the inhalation route for a sub-acute duration (28 days) and sub-chronic duration (90 days) respectively, and to provide robust data for quantitative inhalation risk assessments [4, 5]. Data generated from OECD TG 412 and OECD TG 413 are considered acceptable by regulatory agencies to conclude on the potential possible adverse toxicological effects likely to arise from repeated inhalation exposure to a substance. Furthermore OECD TG 451 can be used to generate data appropriate to assess the long-term carcinogenicity of a substance. However conducting an in vivo study according to OECD TG is a time and resource intensive undertaking with ethical implications due to the number of animals required. The aim of this case study is to provide a proof-of-concept demonstration of how the GRACIOUS Framework can allow application of grouping and read-across to support the streamlined hazard assessment of similar MWCNT and reduce the overall burden of testing.

The user enters the GRACIOUS Framework by inputting basic information related to the purpose for grouping, context under consideration and basic physicochemical (PC) characteristics of the NFs which allows appropriate pre-defined grouping hypotheses to be identified if available [1]. An accompanying Integrated Approach to Testing and Assessment (IATA) guides the user in gathering the evidence required to accept or reject a grouping hypothesis. The IATA is a decision tree consisting of a series of decision nodes. However, to answer each decision node hard threshold values are difficult to define, often because data is lacking. In addition, if a value measured is close to a threshold, decision making can be difficult to justify. This paper therefore adopts a quantitative method for assessing the similarity of the different materials in the proposed group which is then interpreted by expert analysis, reducing the need to depend upon thresholds at each decision node.

The initial step in the implementation of the IATA for grouping is a review of existing literature to identify relevant existing information that might be of a sufficient quality to allow a grouping decision to be made. Integration of existing information may allow the burden of testing to be reduced as further experimentation is only required to fill specific data gaps. If experimental testing is required, guidance is provided on what testing should be performed through an IATA-specific tiered testing strategy, where Tier 1 focuses on simple in vitro, in silico or *in chemico* protocols, Tier 2 moves onto more complex in vitro models (including 3D co-culture and repeat exposures) and Tier 3 includes the use of in vivo methods. The choice of tier at which a user exits the IATA reflects the initial purpose for grouping and the associated level of uncertainty considered acceptable for the user’s needs. Grouping and read-across for regulatory purposes will require a significant degree of scientific justification therefore evidence up to and including Tier 3 in vivo data should be considered.

The GRACIOUS framework has six pre-defined grouping hypotheses related to the inhalation route of exposure, four of which deal with potential fate and hazard outcome for NFs differentiated by dissolution rate and potential drivers of hazard related to the release of toxic ions and/or biopersistence [6]. Two further hypotheses for specifically grouping HARNs based on their potential to cause lung disease or mesothelioma after inhalation exposure have been developed [7] (Table 1).

**Table 1:** Gracious pre-defined hypotheses for inhalation route of exposure

Hypothesis title	Reference
Respirable NFs with an instantaneous dissolution rate: Following inhalation exposure, the toxicity is driven by and is therefore similar to those of the constituent ions or molecules	[6]
Respirable NFs with a quick dissolution rate: Following inhalation exposure both NFs and constituent ions or molecules may contribute to toxicity, but there is no concern for accumulation. Toxicity (also) depends on the location of the ionic or molecular release	[6]
Respirable NFs with a gradual dissolution rate: Following inhalation exposure both NFs and constituent ions or molecules may contribute to toxicity and there is some concern for accumulation. Toxicity (also) depends on the location of the ionic or molecular release	[6]
Respirable NFs with a very slow dissolution rate: Following inhalation exposure, toxicity is driven by the NFs and accumulation of NFs in the lungs can lead to long-term toxicity	[6]
Respirable, biopersistent, rigid HARN: Following inhalation exposure, long-term pulmonary retention of HARN can occur resulting in lung toxicity	[7]
Respirable, biopersistent, rigid HARN: Following inhalation exposure and translocation of HARN to the pleura, mesothelioma development can occur	[7]

The HARN IATA to support these grouping hypotheses are based upon the structure–activity relationship governing asbestos carcinogenicity following inhalation, which defines the parameters driving the toxicokinetics from inhalation exposure to retention at the target tissue and the biological interactions leading to an adverse outcome [8]. Each GRACIOUS grouping hypothesis is endpoint specific; the HARN hypotheses deal with the potential hazard posed by fibres within the lung which may lead to chronic inflammation, fibrosis (e.g. asbestosis) or tumour formation, and the hazard posed to the mesothelial tissue surrounding the lung. Inhalation exposure to asbestos is causatively linked to the development of a tumour of the mesothelium, mesothelioma, in exposed populations [9]. Based on the fibre pathogenicity paradigm [8], the HARN IATA prompts users to address relevant questions regarding the morphology, biopersistence and inflammatory potential of the HARNs under investigation to support the formation of a group [7]. The rationale for the selection and justification for inclusion of specific parameters and thresholds for inclusion and exclusion in a group according to the HARN IATA are outlined in detail in [7]. The toxicity of several MWCNTs has been assessed in vitro and in vivo. Existing data can therefore be used as a case study to test whether robust groups can be formed using the HARN IATA. Existing data can also be used to identify whether in vitro outcomes are predictive of in vivo responses.

The objectives of this case study are to use the GRACIOUS HARN IATA to:

- Determine whether the MWCNT under investigation can be grouped based on common fate and hazard potential.
- Identify data-rich members of the group (i.e. data generated according to relevant OECD TG) to select potential source material(s) for read-across.
- Determine whether members of group(s) are sufficiently similar to support interpolation of data from the source to target MWCNT.
- Determine if in vitro models provide a good prediction of in vivo outcomes.

However, it is worth noting, that even if the MWCNTs investigated are all hazardous, it is possible that they are not sufficiently similar in their hazard potential and mechanism of hazard to form a single group. This case study therefore provides an opportunity to assess the ability to make and justify such decision making.

## Methods

### MWCNT panel

The MWCNT under investigation comprise a panel assembled by National Research Centre for the Working Environment, Copenhagen (NRCWE) (Table 2) and represent a range of morphologies and surface chemistries. The characterisation and hazard assessment of this panel were reported in a number of peer-reviewed publications [10–13] (Additional file 1: Table S1).

**Table 2:** Panel of MWCNT included in case study

**MWCNT**	**Manufacturer/Distributor**	**Product Code**	**Surface Chemistry**	Carbon (%)	**Length**		**Diameter**		**Aspect Ratio **	**Surface Area**	**Oxygen Content**
					Mean (µm)	± SD (µm)	Mean (nm)	± SD (nm)	**(L/D)**	(m^2^/g)	mmol/g
NM-400	JRC	JRCNM04000a	PRISTINE	86.2	0.85	0.1	11	3	77.27	254	0.79
NM-401	JRC	JRCNM04001a	PRISTINE	99.7	4	0.37	67	24	59.7	18	0.03
NM-402	JRC	JRCNM04002a	PRISTINE	96.1	1.4	0.19	11	3	127.27	226	0.28
NM-403	JRC	JRCNM04003a	PRISTINE	99.1	0.4	0.03	12	7	33.33	135	0.19
NRCWE006	Mitsui/Hadogaya	XNRI MWNT-7	PRISTINE	99.6	5.7	0.49	74		77.02	26	0.08
NRCWE040	Cheaptubes	sku-030102	PRISTINE	98.6	0.52	0.59	20.56	6.9	25.29	150	0.35
NRCWE041	Cheaptubes	sku-030202	OH	99.2	1	2.95	26.38	11.1	37.9	152	1.69
NRCWE042	Cheaptubes	sku 030,302	COOH	99.2	0.72	0.97	20.5	5.32	35.12	141	4.09
NRCWE043	Cheaptubes	sku 030,107	PRISTINE	96	0.771	3.471	26.73	6.88	28.84	82	0.18
NRCWE044	Cheaptubes	sku 030,207	OH	96	1.33	2.454	32.55	14.4	40.92	74	0.23
NRCWE045	Cheaptubes	Sku 030,307	COOH	92	1.553	2.954	28.07	13.85	55.32	119	0.63
NRCWE046	Cheaptubes	sku-030111	PRISTINE	98.7	0.72	1.2	17.2	5.8	41.8	223	0.63
NRCWE047	Cheaptubes	sku 030,112	OH	98.7	0.53	0.59	12.96	4.4	40.89	216	0.26
NRCWE048	Cheaptubes	sku030113	COOH	98.8	1.6	5.6	15.08	4.7	106.1	185	0.58
NRCWE049	Cheaptubes	sku 030,114	NH_2_	96	0.731	1.473	13.85	6.09	52.77	199	0.33

PC characterisation data extracted from [11, 13]. Surface area determined by Brunauer–Emmett–Teller (BET) surface area analysis, Carbon purity and oxygen content determined by combustion elemental analysis, length and diameter measured by scanning electron microscopy of MWCNT dispersed by ultrasonication in media containing 2% fetal calf serum

### Application of the IATA

**Fig. 1:**
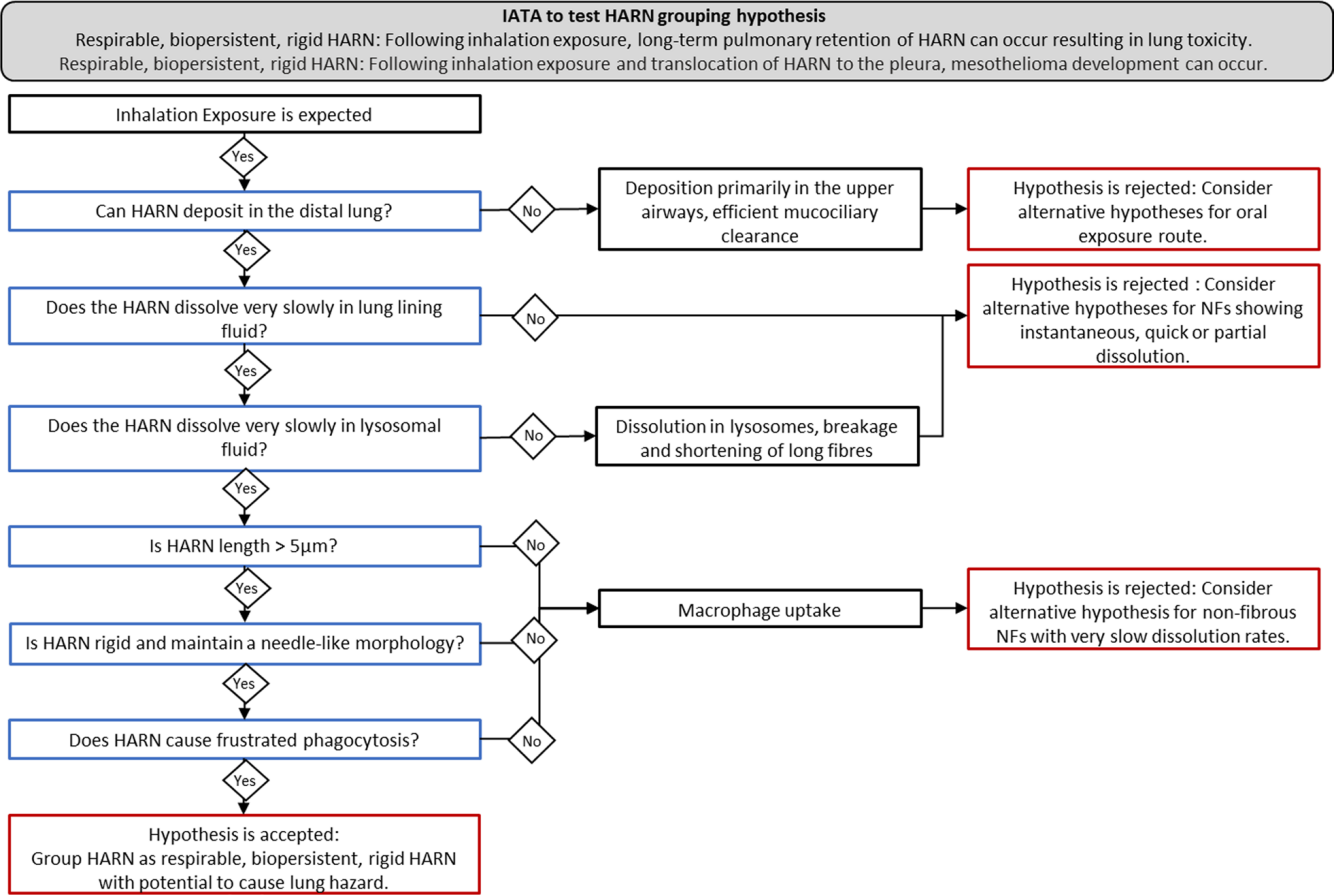
Integrated Approach to Testing and Assessment (IATA) to test the GRACIOUS pre-defined grouping hypothesis: Respirable, biopersistent, rigid HARN: Following inhalation exposure, long-term pulmonary retention of HARN can occur resulting in lung toxicity. Adapted from [7]

Application of the HARN IATA for this case study followed a 2-step process. For reference, the HARN IATA decision tree and tiered testing strategy are presented in Fig. 1 and Table 3, respectively [7]. Relevant information to support grouping of the MWCNT panel was initially collected according to the IATA from the data presented in the studies conducted at NRCWE, termed the core data set (Additional file 2: Table S2). Input of data into a data matrix was followed by an initial assessment of whether the grouping hypotheses could be accepted or rejected. A targeted literature search was subsequently conducted whereby data relevant to each decision node of the IATA was identified in the wider literature for 2 MWCNT, for which the grouping hypothesis was initially accepted. This wider data set along with generation of new data was used to fill the specific data gaps required to confirm acceptance of grouping hypotheses for the MWCNT selected in the initial assessment. Integration of additional data into the data matrix allowed the degree of similarity between grouped and selected ungrouped MWCNT to be assessed.

**Table 3:** Tiered Testing Strategy to support the HARN IATA. Recommended methods and models to address each IATA decision node are set-out according to the appropriate tier of testing. Adapted from [7]

Can HARN deposit in the distal lung?	Does the HARN dissolve very slowly in lung lining fluid (pH7.4)?	Does the HARN dissolve very slowly in lysosomal fluid (pH4.5)?	Is HARN length > 5 µm?	Is HARN rigid and maintain a needle-like morphology?	Does HARN cause frustrated phagocytosis?
Tier 1					
Review existing data sets					
Estimation of aerodynamic diameter from diameter and density of HARN	Batch dissolution test or dissolution in continuous flow system in lung lining fluid	Batch dissolution test or dissolution in continuous flow system in lysosomal fluid	Length distribution profile from TEM/SEM images	Mean diameter of HARN from TEM/SEM. Include supporting images	Inflammasome activation in macrophage exposed to HARN: IL-1β release
Tier 2					
Review existing data sets					
Measurement of mass median aerodynamic diameter (MMAD) from airborne dispersion of material		Durability in cellular systems	HARN length measurement from airborne dispersion of material from TEM/SEM images	HARN size measurement from airborne dispersion of material from TEM/SEM images. Include supporting images	In vitro granuloma formation
Tier 3					
Review existing data sets					
Quantification of lung deposition during in vivo inhalation studies	Quantification of lung burden and clearance kinetics during in vivo inhalation studies			Fibre rigidity of HARN demonstrated by morphological assessment and/or size measurements after incubation with macrophages in vitro or in vivo	Evidence of frustrated phagocytosis and granuloma formation from in vivo exposure (OECD GD 39)

### Experimental work

#### DCFH_2_-DA

Acellular detection of reactive oxygen species (ROS) production was assessed using the 2'-7'-dichlorodihydrofluorescin diacetate (DCFH_2_-DA) probe according to the GRACIOUS standard operating protocol (Boyles et al. 2021). DCFH_2_-DA was chemically hydrolysed by incubation with 0.01 M NaOH, neutralized and diluted to 10 µM DCFH_2_ in phosphate-buffered saline. During this reaction, test particles were prepared by suspension in phenol red-free minimum essential medium with 2% FCS at a concentration of 1000 µg/ml, followed by ultra-sonication in a water bath and serial dilutions performed to obtain a concentration range of 16, 31, 62.5, 125 µg/ml. Each treatment was then added, in triplicate to a 96-well plate at a volume of 25 µl, followed by addition of 225  µl 10 µM DCFH_2_ to each well. Final MWCNT concentrations of 1.6, 3.1, 6.25, 12.5 µg/ml were obtained, which were incubated at 37 °C for 90 min. After this time, samples were centrifuged at 3000 × g for 15 min, and 100 µl of each well was moved to a black 96-well plate to read fluorescence at ex/em wavelengths of 485/530 nm.

### Inflammasome activation

The human monocyte cell-line THP-1 (TIB-202, ATCC) was differentiated into macrophages by adding 100 ng/ml phorbol 12-myristate 13-acetate (PMA) to 5 × 10^5^ cells/ml and plating 100 µl of the cell suspension in each well of a 96-well plate. The THP-1 cells were incubated with PMA for 3 h which allowed the cells to attach to the wells. After 3 h, the medium was removed, and fresh culture medium was added, and the cells were incubated at 37 °C for 20–24 h. Next, the MWCNT (NM-401 and NRCWE006) were added to the wells in concentrations ranging from 1.56 to 100 µg/ml. The cells were incubated for 24 h at 37 °C. MWCNT were dispersed by water-bath sonication in cell culture media prior to administration. Light microscopy images of MWCNT suspensions directly after preparation and MWCNT deposition onto THP-1 cells after 24 h incubation are provided in Additional file 5: Fig. S1. Supernatant was removed and IL-1β production was measured using ELISA according to manufacturer's instructions (R&D DuoSet, R&D Systems, MN USA).

### Similarity assessment

Pairwise similarity assessment was conducted between NM-401, NRCWE006, NM-403 and NRCWE040 according to Tier 1 PC characterisation and hazard assessment data to assess similarity between the 2 grouped MWCNT (NM-401 and NRCWE006) and compare with 2 MWCNT which rejected the grouping hypothesis (NM-403 and NRCWE040) [14]. As the data was largely extracted from the published literature the similarity assessment was based on the summary metrics provided rather than raw data e.g. mean ± SD for fibre length, fold-change over control for mRNA expression. A summary metric was selected to represent each decision node endpoint. PC characteristics were represented by mean values whereas hazard data was represented by values obtained at maximum dose/timepoint assessed (when demonstrated to be sub-lethal), normalised to negative control for each assay, or EC_50_ value. The fold difference between source to target was obtained by dividing the higher value with the lower value in order to obtain a score > 1. Interpretation of the level of similarity observed and justification to support read-across was provided by expert opinion. The relative level of similarity was assessed taking into account the magnitude of change over negative control and magnitude of response measured in comparison to a representative test material (RTM) when included in an assay.

## Results

### Entry to the framework-selection of hypothesis

Identification of an elongated structure (Aspect ratio > 3) in the Basic Information step of the GRACIOUS Framework [1] selects the HARN grouping pre-defined hypotheses as most relevant for this panel of MWCNT.

The HARN IATA designed to test both HARN grouping hypotheses prompts users to address relevant questions regarding the deposition, biopersistence, morphology and inflammatory potential of the HARNs to support grouping according to the potential to pose a similar fibre-like hazard (Fig. 1). The specific data endpoints required to address each decision node of the IATA is set-out in the tiered testing strategy (Table 3). In the first instance, it is recommended that existing data is used. If existing data is not available the tiered testing strategy can also guide the user in the appropriate methodology to fill data gaps.

The data required to answer questions posed in each decision node of the IATA for the MWCNT panel was extracted from the NRCWE studies [10–13] and inputted into the IATA data matrix at the appropriate tier. An overview of relevant data availability from the NRCWE data set is included in Table 4. A summary of data gathered from the NRCWE studies according to each IATA decision node is presented in Table 5, alongside the IATA outcome based on this data set. The complete IATA data matrix table including values and methods from the NRCWE data set for each decision node at Tier 1 is included in Additional file 2: (Table S2). No Tier 2 data and only qualitative Tier 3 data was identified in the NRCWE data set.

**Table 4:** Overview of the data availability for the MWCNT panel from the data set extracted from the NRCWE studies for each IATA decision node and Tier of the tiered testing strategy

	Can HARN deposit in the distal lung?	Does the HARN dissolve very slowly in lung lining and/or lysosomal fluid?	Is HARN length > 5 µm?	Is the HARN rigid and maintain a fibrous, needle-like morphology?	Does the HARN cause frustrated phagocytosis?
Tier 1	Estimated D_ae_ based on mean diameter and assumed density of not appropriate for heterogenous HARN	No data from in vitro dissolution studies. MWCNT predicted to be durability based on graphene structure	Size distribution profiles not available. Mean length reported	Diameter of primary particle reported plus supporting SEM images where available	Frustrated phagocytosis not specifically addressed
Tier 2	Measured MMAD not reported	No data from intracellular dissolution studies	Size distribution of aerosolized MWCNT not reported	Size distribution of aerosolized MWCNT not reported	No data from 3D granuloma model
Tier 3	Deposition after inhalation exposure not reported	Qualitative evidence of biopersistence in lung was available		Qualitative evidence of fibre rigidity for NM-401 and NRCWE006 in BAL macrophages and lung and liver tissue was available	Qualitative evidence of frustrated phagocytosis of NM-401 and NRCWE006 in BAL macrophages was available

Please note that this is not a summary of all data for all HARN publications available to date, only those relevant to this case study

**Table 5:**
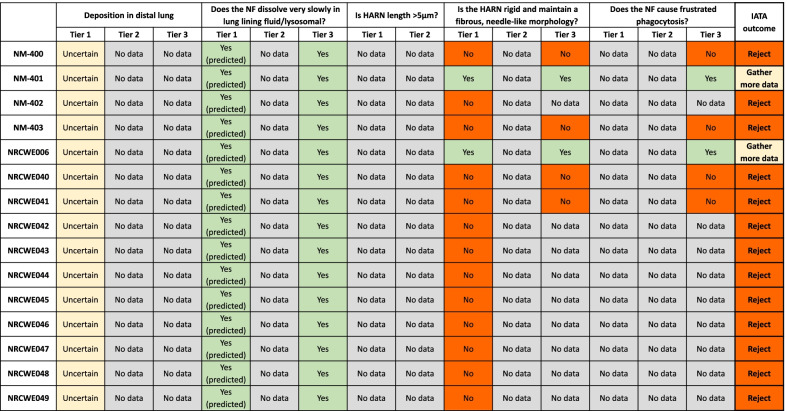
Summary of IATA results for MWCNT panel according to each IATA decision node and data available from NRWCE studies at each tier of testing

Yellow box: Decision node outcome is uncertain due to gaps or limited confidence in available data, Grey box: No relevant data available, Green box: Yes to decision node question, Red: No to decision node question

Analysis of the core data set revealed that for 13 MWCNT the grouping hypothesis could be rejected based on the currently available NRCWE data alone. The hypothesis was rejected due to failure of the 13 MWCNT to meet the IATA Tier 1 threshold for rigidity (diameter > 30 nm). To ensure the justification for grouping is sufficiently stringent the MWCNT must meet the criteria of each IATA decision node to be included in the group. Robust evidence that the MWCNT do not meet the criteria of one decision node is sufficient for that MWCNT to be excluded from the group. SEM and TEM images showed these MWCNT have a curled morphology and present as tangled agglomerates rather than straight fibres, confirming the lack of rigidity of these MWCNT (Additional file 6: Fig. S2, Additional file 9: Fig. S3). A lack of rigidity and failure to cause frustrated phagocytosis was further qualitatively demonstrated by microscopy images of 4 of these MWCNT (NM-400, NM-403, NRCWE040, NRCWE041), fully engulfed by macrophages isolated from broncho-alveolar lavage (BAL) fluid after in vivo exposure in mice, providing Tier 3 evidence to decline this decision node [10]. Further light microscopy images of the panel of MWCNT confirming the absence of fibre-like morphology of the majority of the panel were produced here (Additional file 10: Fig. S4). Although these 13 MWCNT should not be included in a group based on common fate/hazard potential dictated by fibre morphology, alternative modes of action leading to toxicity should be considered. In this case the user will be prompted to re-enter the GRACIOUS Framework to identify alternative relevant pre-defined hypotheses to support grouping of these HARN.

The grouping hypothesis was not rejected for NM-401 and NRCWE006 based on the currently available information provided within the NRCWE core data set. However, due to the number of data gaps identified in this data set (Table 5) further Tier 1, Tier 2 and Tier 3 data was sought from the literature to support the acceptance of the HARN grouping hypotheses and strengthen the justification of group formation. A targeted literature search was conducted, restricted to studies utilising these two specific MWCNT and focused on filling the identified data gaps (Additional file 3: Table S3). In addition, experimental testing was performed to fill data gaps not addressed by published literature. Evidence collected through application of the IATA to this wider data set and de novo data generated following the tiered testing strategy were integrated with the data matrix with the core data set (Table 6). A summary of the resulting decision node outcomes is presented below.

**Table 6:** IATA outcome for NM-401 and NRCWE006 after integration of additional decision node information gathered from targeted literature search

	Deposition in distal lung			Does the NF dissolve very slowly in lung lining fluid/lysosomal?			Is HARN length > 5 µm?		Is the HARN rigid and maintain a fibrous, needle-like morphology?			Does the NF cause frustrated phagocytosis?			IATA outcome
	Tier 1	Tier 2	Tier 3	Tier 1	Tier 2	Tier 3	Tier 1	Tier 2	Tier 1	Tier 2	Tier 3	Tier 1	Tier 2	Tier 3	
NM-401	Uncertain	Yes	Yes	Yes (predicted)	No data	Yes	No data	Yes	Yes	No data	Yes	Yes	No data	Yes	Accept grouping hypothesis
NRCWE006	Uncertain	Yes	Yes	Yes (predicted)	No data	Yes	Yes	Yes	Yes	No data	Yes	Yes	Yes	Yes	Accept grouping hypothesis

### Can HARN deposit in the distal lung?

The recommended Tier 1 method to address this decision node is to estimate the aerodynamic diameter (D_ae_) of a HARN based on measured HARN diameter and density. However this approach may not be sufficiently robust for heterogenous HARN with wide size distribution profiles such as MWCNT [7]. Therefore, to answer this decision node required escalation to Tier 2 testing. The mass median aerodynamic diameter (MMAD) of NM-401 and NRCWE006 measured from an airborne dispersion prepared for inhalation exposure have been reported [15, 16]. Using a cascade impactor the MMADs for both MWCNT were measured as below the decision node threshold of 4 µm, therefore the outcome for decision node ‘*Can HARN deposit in the distal lung?*’ was YES for both NM-401 and NRCWE006. Confirmation that both MWCNT will deposit in the distal regions of the lung upon inhalation exposure (Tier 3) was further demonstrated in in vivo inhalation studies in mice [17] and rats [15, 18–20].

### Does the NF dissolve very slowly in lung lining fluid/lysosomal fluid?

The testing strategy recommends that in the first instance (Tier 1 testing) dissolution of HARN is assessed acellularly. No data is available from acellular dissolution studies for NM-401 in the published literature. NRCWE006 was included in an acellular dissolution study and showed significant (~ 10%) mass loss after a 3 week incubation in Gambles solution (pH 4.5) [21]. However, a progressive loss of mass was not observed over the entire time-course of the experiment, up to 24 weeks. As MWCNT are generally predicted to be durable based on the stability of the graphenic structure of the MWCNT backbone [22], the paper’s authors suggest the initial loss of mass may be due to the presence of surface defects which could act as points of weakness for chemical attack and resulting in partial breakdown of the MWCNTs, however this was not further explored [21]. The decision node outcome was considered UNCERTAIN at Tier 1. Due to the ambiguous results for Tier 1 further information from higher tier studies was sought. No Tier 2 data (dissolution rate in a cellular system) was available in the published literature for either NM-401 or NRCWE006 instead extensive evidence of long-term biopersistence in in vivo (Tier 3) models was identified for both MWCNT [12, 15, 17, 19]. Therefore the outcome of the decision node ‘*Does the NF dissolve very slowly in lung lining fluid/lysosomal fluid?’* was concluded to be YES for both MWCNTs.

### Is HARN length > 5 µm?

Tier 1 requires a size distribution profile for the HARN samples and the percentage of fibres > 5 µm in length to be reported. The NRCWE data set only reports a mean fibre length for NM-401 and NRCWE006, 4.0 ± 0.37 µm and 5.7 ± 0.49 µm, respectively [11], indicating both samples are close to the 5 µm threshold. However as the IATA does not consider a summary metric reporting median or mean fibre length sufficient to meet the criteria for this decision node, additional information on the size distribution profiles of both MWCNT is required to determine the outcome for this decision node. A number of studies provide size distribution profiles for NRCWE006 reporting > 10% of HARN are > 5 µm in length [23–25]. The decision node outcome is therefore YES for NRCWE006 at Tier 1. No studies reporting the breakdown of length distribution of NM-401 were identified. However, Gaté et al., [15] included representative TEM images of NM-401 from aerosols administered to rats in an inhalation study, which show the predominance of fibres > 5 µm in length within the sample. This evidence of long NM-401 HARN was considered sufficient to support a decision node outcome of YES. Furthermore the observation of needle-like fibres > 5 µm in length within the lung tissue post-inhalation exposure provides additional qualitative evidence to answer decision node ‘*Is HARN length* > *5 µm?’* as YES.

### Is the HARN rigid and maintain a fibrous, needle-like morphology?

According to the diameter measurements of the MWCNT provided in the NRCWE data set, both NM-401 and NRCWE006 met the threshold diameter of 30 nm [11], (67 and 74 nm, respectively). Therefore the outcome of this decision node is recorded as YES. This conclusion was further supported by the provision of representative SEM images from which the measurements were made showing the predominance of straight, needle-like fibres in both MWCNT samples. Additional supporting evidence was identified from in vivo (Tier 3) studies which confirms the Tier 1 outcome of rigidity and demonstrated that both MWCNT maintained needle-like morphology when in contact with cells and tissue from in vivo studies [10, 12, 13].

### Does the NF cause frustrated phagocytosis?

**Fig. 2:**
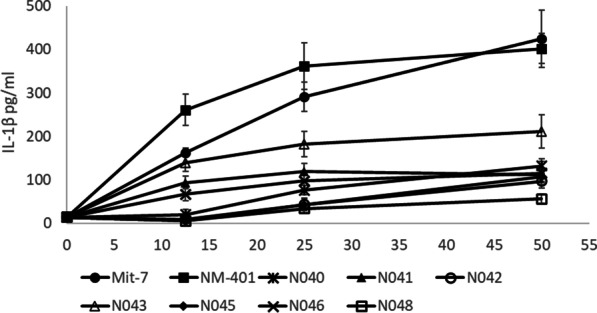
IL-1*β* release from differentiated THP-1 cells exposed to source (NRCWE006) and target (NM-401) MWCNT for 24 h measured by ELISA (*n* = 3). NRCWE040, NRCWE041, NRCWE042, NRCWE043, NRCWE045, NRCWE046 and NRCWE048 are included for comparison with MWCNT which are not included in the group

Data in the literature was not available for both MWCNT with respect to frustrated phagocytosis and so de novo data was generated according to Tier 1 of the tiered testing strategy. A number of the MWCNT already excluded from the proposed group were included in this study to contextualise the response elicited by NRCWE006 and NM-401 and test the robustness of the decision node for grouping HARN based on a common mechanism of toxicity. Exposure of the human monocytic cell line THP-1 to NM-401 and NRCWE006 led to induction of a concentration-dependent release of IL-1β protein (Fig. 2). This data confirms previous reports of NRCWE006-mediated NALP3 activation and IL-1β release due to lysosomal disruption [26–28]. The decision node ‘*Does the NF cause frustrated phagocytosis*?’ was determined to be YES for both MWCNT. A number of the MWCNT caused a slight increase in IL-1β release however to a much lesser degree than NRCWE006 or NM-401. The decision node outcome was further supported by qualitative microscopy evidence of both NM-401 and NRCWE006 causing lysosomal disruption (intracellular ‘vesicle escape’ visualised by TEM, [29]) and frustrated phagocytosis in macrophages both in vitro (Tier 1) and in vivo (Tier 3) [10].

### IATA outcome

Evidence from additional studies strengthened the grouping of NM-401 and NRCWE006 by providing sufficient data related to deposition in the distal lung, biopersistence in lung tissue, fibre length and morphology, and activation of frustrated phagocytosis (Table 6). The grouping hypothesis is therefore accepted for NM-401 and NRCWE006.

### Progression to similarity assessment

**Fig. 3:**
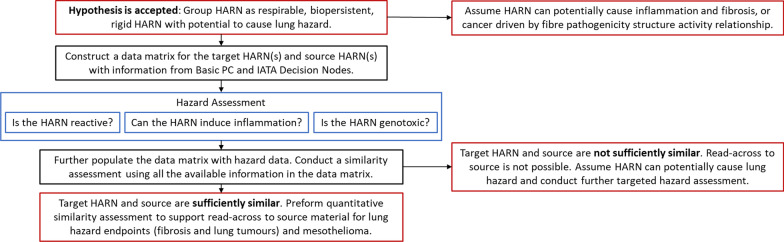
Guidance on how to progress to similarity assessment after initial grouping according to the pre-defined HARN hypothesis is accepted

Once a group has been formed a similarity assessment between group members can be conducted if required for the user’s purpose e.g. to build an argument for read-across between group members. Here we compared the data collected for NM-401 and NRCWE006 according to the basic PC characterisation of the MWCNT panel and available Tier 1 hazard assessment data to assess the degree of similarity between the grouped MWCNT. Inclusion of additional hazard endpoints allows the mechanistic underpinning of the group to be strengthened. Therefore, assessment of the similarity in hazard outcomes between group members, focussing on MWCNT reactivity, inflammatory potential and genotoxicity, were included (Fig. 3). For hazard assessment, only data from literature where the MWCNT were directly compared using the same experimental set-up were included.

### Similarity assessment

Evidence of acceptable levels of similarity between group members may be used to build a read-across argument for interpolation of hazard data between a source and target material. Given the wealth of information available from hazard assessment of NRCWE006 which includes OECD TG inhalation studies and led to an IARC classification of this MWCNT as a Group 2B (possible human carcinogen)[30], NRCWE006 could be considered a data-rich source material for the HARN group. The aim of this similarity assessment is to determine whether a sufficient degree of similarity can be evidenced to justify the interpolation of hazard data from long-term inhalation studies (such as OECD TG 413: [18], OECD TG 451: [19]) from NRCWE006 (source) to NM-401 (target) for which such data is lacking.

The similarity assessment between NRCWE006 and NM-401 was based on the pairwise difference (fold-change) of a summary metric selected as a representative output for each decision node endpoint measured at Tier 1 (Table 7). Interpretation of the degree of similarity between HARN was aided by considering the dynamic range of each assay and relative strength of signal generated by each MWCNT compared to the negative control and RTM where included [14]. Furthermore, 2 MWCNT (NM-403, NRCWE040) for which the grouping hypothesis was rejected were included in a pairwise similarity assessment with the source HARN (NRCWE006) to further aid in the interpretation of an acceptable level of similarity to support read-across between the grouped HARN (NRCWE006 and NM-401).

**Table 7:**
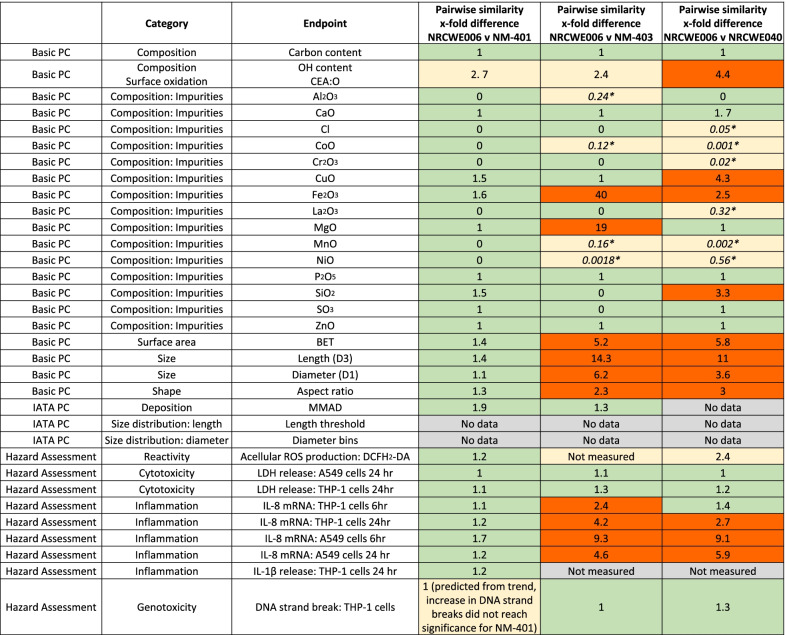
Similarity assessment between source (NRCWE006) and target (NM-401) MWCNTs as well as two MWCNTs for which the original grouping hypothesis was rejected

These two MWCNTs were used to assess the level of similarity required to support grouping and read-across (NM-403, NRCWE040) MWCNT. Data matrix tables were constructed and populated with relevant data from the literature to conduct the pairwise similarity assessment including basic PC characterisation, the endpoints specified by the IATA and hazard assessment endpoints related to reactivity, inflammation and genotoxicity. *For composition: impurities where the contaminant was below the level of detection for the source NRCWE006, the measured value for each target MWCNT is included (% wt, Additional File 8, Table S6).Green box: fold-difference < 2, MWCNT considered similar, yellow box: fold-difference > 2, judgement of similarity requires further consideration, red box: fold-difference > 2 MWCNT considered not similar

## Basic physicochemical characteristics

### Composition

Both NRCWE006 and NM-401 were produced by the CVD method; high levels of purity (Carbon content > 99% for both) and low level of metal impurities (below the limit of detection for many of the metal oxides included) have been reported for both. In comparison NM-403 had 40-fold greater levels of Fe and 19-fold greater levels of Mg contamination when compared to NRCWE006, suggesting mechanisms of toxicity triggered by metal contamination may account for potential hazard responses to NM-403 but not for NRCWE006 (or NM-401). Where the contaminant being measured was below the limit of detection for the source NRCWE006, but not the target MWCNT a pairwise comparison could not be conducted. The measured value of metal (% wt) is included in Table 7, indicated by an asterisk, to highlight the level of dissimilarity between the source and target. For example NRCWE040 is contaminated with 0.56% wt NiO, which could not be detected in NRCWE006. NRCWE006 has 2.7-fold greater content of OH than NM-401. However this level of dissimilarity can be contextualised by comparison between NRCWE006 and a MWCNT modified by oxidation (NRWCE042, Table 2) which shows a 50-fold difference in oxygen content. Therefore, both NM-401 and NRCWE006 (as well as NM-403 and NRCWE040) are similarly designated as unfunctionalized MWCNT.

### Size and shape

Both NRCWE006 and NM-401 are relatively thick MWCNT with a mean diameter in a comparable range (1.1-fold difference) reflected by a specific surface area differing by only 1.44-fold. Conversely, NM-403 and NRCWE040 are considerably thinner with mean diameters 6 and 3.5-fold smaller than the source material NRCWE006 and 5.1 and 5.7-fold greater surface areas, respectively. Qualitative EM images confirm the differences in diameter and impact this has on HARN morphology as NRCWE006 and NM-401 appear as thick, straight, needle-like fibres whereas NM-403 and NRCWE040 have a thinner and curled appearance (Additional file 6: Figure S2), forming tangled agglomerates. The difference in mean length between the target and source MWCNT samples is less than 2 µm (1.4-fold difference), whereas NM-403 and NRCWE040 are on average much shorter than NRCWE006 showing a 14- and tenfold difference, respectively. Interestingly, the mean lengths of NRCWE006 (5.7 ± 0.49 µm) and NM-401 (4 ± 0.37 µm) straddle the threshold set by the IATA to differentiate a HARN likely to pose an asbestos-like fibre hazard from a HARN sample that will not conform to the fibre pathogenicity paradigm. A comparison of the size profiles of each sample will therefore be required to strengthen the conclusion that both MWCNT may pose a hazard driven by the same mechanism of action. Based on the current available data NM-401 and NRCWE006 are considered to show an acceptable similarity in terms of length and morphology to support rather than reject the basis of the grouping. Furthermore as NM-401 has a shorter mean fibre length than NRCWE006, read-across can be considered appropriate for hazard outcomes driven by exposure to long fibres (> 5 µm) which represents a worse-case scenario.

As a result of their comparable mean diameters and length, the fold-change difference in average aspect ratio (AR) between NRCWE006 and NM-401 MWCNT remains close to 1, suggesting they are similar. However NM-403 and NRCWE040 show a 2–threefold difference in AR compared to the source material and NM-401. AR is derived from diameter and length and is not an independent metric. The same AR can represent a number of combinations (high AR of an extremely thin but short HARN may similarly represent a thick but long fibre), therefore it is not considered an appropriate measure of similarity.

### IATA Criteria

#### Deposition

The likelihood that each MWCNT will deposit in the distal lung upon inhalation is roughly predicted from evidence that the HARN has an aerodynamic diameter (D_ae_) of < 4 µm as this represents the respirable fraction of particles that reach the alveoli (d50 = 4 μm) [31].

Due to heterogenicity evident within the MWCNT samples it was not appropriate to estimate the D_ae_ as suggested at Tier 1 of the IATA for this decision node, but rather escalate directly to Tier 2 and measure the mass median aerodynamic diameter (MMAD). NRCWE006, NM-401 and NM-403 have measured MMADs below the 4 µm threshold and less than twofold difference between each other which would suggest they may behave similarly when inhaled (data not available for NRCWE040). To predict whether these MWCNT would follow the same toxicokinetic pathway within the lung upon inhalation exposure would require further modelling of deposition. The MMAD can be utilised to facilitate comparisons between the predicted deposition patterns of inhaled particles as modelled by lung deposition modelling software such as the Multiple Path Particle Deposition model [32]. However currently the use of computational models of deposition are not considered sufficiently robust to predict the deposition of fibre shaped materials. Therefore, similarity in terms of deposition cannot be adequately judged from this metric. However, the 3 MWCNT have been shown to deposit in the distal regions of the lung in independent inhalation studies, therefore it can be concluded both may pose an inhalation hazard which may results in pathogenic effects in the distal regions of the lung. Although similarity cannot be assessed, no evidence exists that NM-401 would present less of an inhalation hazard than NRCWE006 in terms of deposition efficiency to preclude the use of read-across to predict hazard.

### Dissolution/biopersistence

No acellular dissolution data exists for NM-401 therefore a similarity assessment could not be conducted at Tier 1 for this decision node. The Tier 3 data reported for NM-401 and NRCWE006, which supports grouping of these MWCNT as biopersistent, was however not derived from the same experimental set-up. Therefore, a similarity assessment could not be conducted between the reported clearance kinetics or biopersistence half-lives. However, both NM-401 and NRCWE006 have been shown to persist in the tissue beyond the length of the long-term exposure. It can therefore be concluded that upon exposure in humans both will persist and accumulate in the tissue, thus presenting a similar long-term hazard. NM-403 and NRCWE040 have also been shown to persist in lung tissue up to at least 1 year post exposure [12].

## Hazard assessment

### Reactivity

**Fig. 4:**
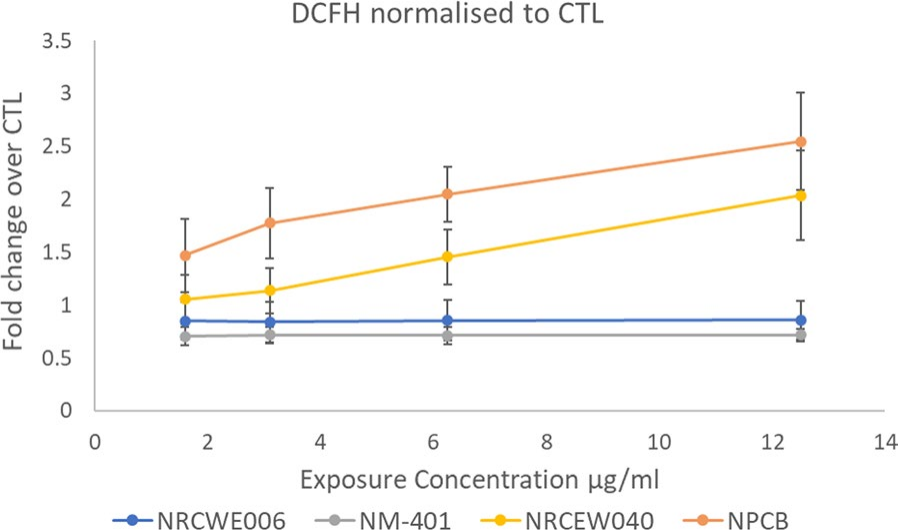
Reactive oxygen species (ROS) generation by source (NRCWE006) and target (NM-401, NM-403, NRCWE040) MWCNT measured by DCFH probe (*n* = 3)

To assess reactivity at Tier 1, intrinsic ROS generation was measured by using the acellular DCFH_2_-DA probe (Fig. 4, Additional file 7: Table S5). No significant ROS generation compared to the control was observed for either NRCWE006 or NM-401 at the concentration range tested ([33]). A slight reduction of signal from negative control was observed for both when the top exposure concentrations were compared, suggesting at this concentration the MWCNT may start to interfere in the assay, blocking or quenching fluorescence detection. Printex 90 carbon black (NPCB), known to produce high levels of ROS [34, 35] was run in parallel as a representative test material (RTM) for the assay and generated a positive ROS signal that was 2.5 times greater than the vehicle control (2.9-fold greater signal than NRCWE006). This data supports the conclusion that no intrinsic ROS generation could be detected for NRCWE006 or NM-401. NRCWE040 generated a dose-dependent increase in ROS signal, which at the top dose showed a 2.3-fold greater signal compared to NRCWE006, which is in line with the positive signal generated by the RTM, NPCB. NM-403 was not included in the reactivity assessment. Given the relative lack of response from NRCWE006 or NM-401, it was concluded that these MWCNTs were similar in that no intrinsic ROS generation could be detected for either.

## Cytotoxicity

Compared to controls, exposure to the highest concentration of NRCWE006 and NM-401 resulted in a slight, but not significant decrease in A549 and THP-1 cell viability as quantified by a nucleo-counter [10]; viability was also unchanged in FE1-MML epithelial cells [11]. When also considering the NM-403 and NCRWE040 MWCNT no difference in cytotoxicity greater than twofold was seen between the MWCNTs. All MWCNTs were considered to be similar in terms of their lack of cytotoxicity in the cells and at the concentration and times employed in vitro*.*

## Inflammation

The highest concentrations of NRCWE006 and NM-401 tested induced an increase in IL-8 mRNA in exposed A549 lung epithelial cells (18-fold and tenfold compared to negative control in the assay after 24 h) and THP-1 differentiated macrophages (28-fold and 43-fold compared to negative control in the assay after 24 h), leading to ≤ 1.8-fold differences between these MWCNT in the pairwise similarity assessment. NM-403 and NRCWE040 both induced an increase in IL-8 mRNA over control albeit to a lesser degree (fourfold and threefold in A549 cells and eightfold and 12-fold in THP-1 cells following 24 h cell exposure). The IL-8 mRNA increase in A549 and THP-1 following exposure for NM-403 and NRCWE040 are relatively similar and much less than the increases observed when exposing the cells for NRCWE006 and NM-401. (between 2.33–fivefold differences) [10]. No positive RTM particle was included in the assay results to judge the relative magnitude of responses reported. In lieu of this crucial data for interpretation of similarity a < twofold change is considered the default acceptable level. Furthermore, newly generated data from THP-1 cells exposed to NRCWE006 and NM-401 shows that both MWCNT stimulate the release IL-1β, indicative of NALP3 activation (1.2-fold difference in measured IL-1β released) (Fig. 2, Additional file 7: Table S6). Taken together, pro-inflammatory responses were considered to be induced with a similar degree of potency by NM-401 and NRCWE006 when MWCNT are compared on a mass dose basis (pairwise differences for each inflammatory endpoint ranged from 0.8 to 1.7).

## Genotoxicity

NRCWE006, NM-403 and NRCWE040 induced approximately a twofold increase in DNA strand breaks in THP-1 macrophages as measured by Comet assay [10]. Although NM-401 did not induce a statistically significant increase compared to control, a trend towards a slight increase in DNA strand breaks was observed when assessing the data in terms of SD fold increases [10]. These results indicate that all four MWCNT may have some potential to induce DNA damage in THP-1 macrophages, although to a low degree. Exposure to NRCWE006 or NM-401 did not cause an increase in DNA strand breaks in A549 epithelial cells. [10].

## Conclusion of similarity assessment

Based on the analysis of the pairwise assessment NM-401 appears to have a similar profile for PC characteristics as NRCWE006, which would predict both MWCNT will pose an inhalation hazard driven by fibre morphology and biopersistence, although the similarity of fibre length distributions remains to be confirmed. In contrast the PC properties of NM-403 and NRCWE040 were not identified as being similar to NRCWE006. A prediction of similar hazard potential as the source, NRCWE006, can therefore not be supported for NM-403 and NRCWE040. The conclusion that NRCWE006 and NM-401 are likely to have similar toxic behaviour based on PC properties is further supported by the results from Tier 1 hazard studies which directly compared the reactivity, cytotoxicity, inflammatory potential and genotoxicity of both MWCNTs within the same experimental studies. A similar pattern of responses was observed across each assay tested for NRCWE006 and NM-401. In contrast the responses stimulated by NM-403 and NRCWE040 deviated from those elicited by NRCWE006. Thus, the similarity assessment suggests that NRCWE006 and NM-401 can be grouped according to the hypothesis as respirable, biopersistent, rigid HARN with the potential to cause lung toxicity upon inhalation exposure, and that NRCWE006 can be used as a source material when performing read-across to address data gaps for NM-401. However, NM-403 and NRCWE040 are not members of the same group and therefore an alternative GRACIOUS grouping hypothesis will have to be tested for these materials.

## *Predictivity of tier 1 similarity assessment for *in vivo* similarity*

To allow a read-across argument to be justified, the prioritisation of the parameters by which similarity is judged should be rationalised. The predictivity of the Tier 1 hazard endpoints for in vivo hazard outcomes reported for the MWCNT under investigation has therefore been briefly reviewed. The inhalation hazard posed by both MWCNT has been assessed in a number of in vivo studies, however only limited studies have included both MWCNTs allowing for direct comparison of responses between NRCWE006 and NM-401 (Summarised in Additional File 4: Table S4). One of the core NRCWE studies included in this case study, Knudsen et al., [12], evaluated histological changes in lung tissue 1 year after a single intratracheal (IT) instillation of a panel of MWCNT including NRCWE006 and NM-401 (54 µg/mouse). Although these MWCNT were both present in the lung and liver tissue at the 1 year time point, no significant histopathological changes compared to vehicle control mice were observed in either NRCWE006 or NM-401 exposed mice. However, NM-403 and NRCWE040 both caused lymphocyte infiltrates and granulomatous aggregates with statistically higher grading than NM-401, NRCWE006 or vehicle control mice. These results could suggest that the Tier 1 in vitro assays utilised by the IATA may not be predictive of in vivo hazard as both NM-401 and NRCWE006 samples induced pro-inflammatory changes in the Tier 1 studies but no toxic effect was observed in vivo. However the lack of histopathological changes observed by Knudsen et al. [12] are somewhat contradictory to an alternative study from [36] who exposed mice to NRCWE006 (36 ± 6 or 109 ± 18 μg/mouse), or NM-401 (26 ± 2 or 78 ± 5 μg/mouse), once a week for four weeks via IT instillation. Both MWCNT types persisted in lung tissue 90 days post-exposure, and induced lung inflammation and fibrosis to similar extents. Furthermore both induced changes in expression levels of genes involved in several pro-carcinogenic pathways although no DNA damage was detected in mice exposed to either MWCNT. This is in keeping with a wealth of evidence from multiple further in vivo studies which have assessed the hazard of NRCWE006 (Mitsui-7) exposed by IT and pharyngeal aspiration and shown significant pro-inflammatory changes [13, 24, 26, 27, 37]. On balance the weight of evidence that NRCWE006 and NM-401 are likely to pose a hazard in the lung is stronger than the contradictory evidence provided in the paper by Knudsen et al. [12], therefore an overall conclusion that both NRCWE006 and NM-401 are likely to present a chronic inhalation hazard was reached.

Studies which use IT instillation as in vivo exposure models are useful to indicate hazard but are not considered physiologically-relevant models of inhalation exposure for regulatory assessment of risk [38]. A number of inhalation exposure studies have been conducted with both NRCWE006 and NM-401 (as well as NM-403), summarised in Additional file 3: Table S3. One study assessing the inhalation exposure to NM-401 and NM-403 has been conducted to date with results reported across 2 publications [15, 39], whereas four separate studies have been conducted for NRCWE006 [17–20]. Overall, these studies suggest inhalation exposure to NRCWE006 and NM-401 can lead to adverse inflammatory and fibrotic changes in the lungs. However, the experimental design of these studies differs in length of exposure, aerosol concentrations, mode of exposure and inclusion of post-exposure recovery timepoints. Therefore, results cannot be directly compared to validate the Tier 1 similarity outcomes.

Short-term exposure studies to both NRCWE006 and NM-401 resulted in acute inflammatory and fibrotic changes in the lung which regressed somewhat during the post-exposure recovery period. This may reflect a resolution of pathological outcomes upon the cessation of exposure and removal of pathogenic fibres over time. Long-term exposures as reported in [18] and [19] studies (13 week and 104-week exposure, respectively) appeared to show a dose and duration-dependent effect as pathogenic responses continued to worsen resulting in tumour formation when sufficient dose builds up over time.

Hazard data derived from long-term inhalation studies are used in risk assessment and are prioritised for hazard classifications such as IARC classification of carcinogens. Given the hazard data available for NRCWE006, summarised in (Additional file 3: Table S3), we can refine our case study aim for this similarity assessment to address the specific question:

*Are NRCWE006 and NM-401 considered sufficiently similar to make the prediction that continued exposure to NM-401 for durations and doses reported in the *[18, 19]* studies would results in similar pathogenic responses including fibrosis and tumour formation?*

Here we have gathered evidence to show:

- NRCWE006 and NM-401 can form a hypothesis-based group of HARNs likely to pose an inhalation hazard driven by fibre morphology and biopersistence [7].
- NRCWE006 and NM-401 display similarities in recognised drivers of fibre-hazard i.e. needle-like morphology and length greater than 5 µm. In addition to the similarity in morphology NRCWE006 and NM-401 induced Tier 1 inflammasome activation with similar potency when directly compared in the same experimental studies.
- NRCWE006 and NM-401 have been shown to induce significant, long-term histopathological changes in the lung after exposure by both instillation and inhalation in rats and mice (Table 6, Additional file 2: Table S2, Additional file 3: Table S3).

We therefore propose that an argument could be supported for the use of read-across of long-term hazard data between these 2 MWCNT to allow regulatory hazard data gap filling for NM-401.

## Discussion

### Outcome of grouping

Application of the GRACIOUS Framework to a case study panel of 15 MWCNT supported the acceptance of the pre-defined hypotheses for grouping of 2 MWCNT (NRCWE006 and NM-401) as HARN with predicted similar potential to cause adverse outcomes in the alveolar region and mesothelium after inhalation exposure. The grouping of these MWCNT was substantiated by the IATA-led gathering of evidence to demonstrate these MWCNT display similar characteristics and behaviours recognised as drivers of fibre-related pulmonary disease. Conversely, the 13 MWCNT for which the grouping hypotheses were rejected, failed to meet the IATA criteria. The rejection of 13 of the 15 MWCNT demonstrates the stringency of the is approach in supporting group formation between MWCNT for which only a high degree of similarity in intrinsic characteristics and extrinsic behaviours have been demonstrated, providing a greater degree of confidence in the argument for read-across of data between group members.

A number of alternative methods for grouping MWCNT have utilised supervised and unsupervised modelling approaches to identify selective parameters to differentiate pathogenic from non-pathogenic MWCNT. In a study by Jagiello et al., [40], aspect ratio was proposed as the defining parameter to differentiate and group fibrogenic MWCNT based on the correlation between transcriptional changes leading to lung fibrosis upon exposure to MWCNT with high aspect ratio. The authors developed a QSAR model that utilized the singular parameter of aspect ratio as a predictor of perturbations in key pathways, notably the ‘agranulocyte adhesion and diapedesis’ pathway, in the adverse outcome pathway (AOP) leading to lung fibrosis. However, inhalation exposure to particles may cause fibrosis through a variety of modes of action unrelated to high aspect ratio e.g. pathogenic silica via high surface reactivity and oxidative stress [41]. The selectivity of this QSAR for grouping according to a common mechanism of action is therefore limited. In contrast, the HARN IATA considers similarity across multiple PC parameters to allow grouping of MWCNT underpinned by similarity in mechanism of action leading to an apical adverse outcome e.g. lung fibrosis or mesothelioma. This approach supports a more nuanced sub-grouping of NFs currently categorized by broad common denominators such as “high aspect ratio” by differentiating certain NFs of HARNs which pose a specific hazard from the other variations characterized by attributes which may drive very different toxicity profiles [3].

Fraser et al., [42], utilised computational modelling to assess the various permutations of PC characteristics linked to a panel of toxicity outcomes (cell viability, inflammation, cellular oxidative stress, micronuclei formation, and DNA double-strand breakage) and reported the selective grouping of long and thick MWCNT which was predictive of in vitro hazard. By reporting an outcome similar to the grouping of NM-401 with NRCWE006 presented here, this unbiased approach based on correlating the independent PC inputs with hazard outcomes through principal component analysis further validates the selection of the IATA decision nodes as appropriate for addressing the critical drivers of fibre hazard. Importantly this study also emphasizes the need to use complete size distribution profiles to obtain a robust grouping outcome of heterogenous materials such as MWCNT, in line with GRACIOUS HARN IATA requirement to use % fibre > 5 µm to represent HARN length rather than summary metric.

Focussing on MWCNT genotoxicity, Aschberger et al., [43] evaluated the applicability of chemoinformatics techniques such as hierarchical clustering and principal components analysis to support grouping of MWCNT and read-across genotoxicity hazard data between group members. Although some dose-dependent effects were reported, the overall genotoxicity of the panel of MWCNT under investigation was relatively low and as experimental variation could not be discounted the MWCNT were considered non-genotoxic. Due to the comparable (negative) results, no physicochemical differences were identified which could link genotoxicity to specific differences in the measured PC characteristics and therefore the authors proposed the MWCNT were all members of the same group. As such, the negative results for the genotoxicity endpoints could be read-across from data-rich to data-poor members MWCNT. An alternative QSAR model for predicting the genotoxicity of the same panel of MWCNT under investigation here and included in the Aschberger study was recently developed by Kotzabasaki et al., [44]. In contrast to Aschberger et al., [43] a number of MWCNT from within the overlapping panel were designated genotoxic based on results from in vitro chromosome aberration (micronucleus) assays and the in vivo DNA damage Comet assay. Several supervised and unsupervised in silico models were applied by the authors to predict this genotoxic outcome based on correlations with a panel of PC properties used as input features. The resulting QSAR model predicted genotoxicity using the 3 input features of mean length, % purity and zeta potential [44]. Neither grouping outcome presented by these two studies has been validated for biological relevance, as from the MWCNT panel tested only NRCWE006 has been shown, to date, to cause tumour formation in vivo [19].

Here we show a selective grouping of the MWCNT based on a broader number of hazard endpoints (reactivity, pro-inflammatory changes, genotoxicity), known to be instrumental in the pathogenesis of fibre-related disease including tumour formation in vivo and reflecting the complicated nature of particle and fibre induced disease in human populations. This approach allows for multidimensional grouping to reinforce the mechanistic underpinning of the group and take into account a predicted commonality between group members in both fate and hazard outcomes upon inhalation exposure. We would suggest due to the limited amount of data validating a direct link between specific PC features (e.g. high aspect ratio), [40] and single hazard outcomes (e.g. genotoxicity), [43, 44] currently available, expert-led interpretation of grouping and similarity is still required to substantiate group formation in terms of biological relevance and degree of similarity which may differentiate between MWCNT based on critical drivers of long-term human hazard. Recent work by [45] suggests that in vivo pathological outcomes may be directly linked to the secondary structures and degree of agglomeration of MWCNT which may be predicted from the nominal tube dimensions. This proposal offers a promising approach to grouping MWCNT which could be readily assimilated into the HARN IATA through adaptation of the tiered testing strategy linked to the ‘Is the HARN rigid and maintain a needle-like morphology’ decision node, however a standardised methodology to characterise MWCNT secondary structure which can be influenced by the methods of sample preparation would need to be defined.

## Rejection of the grouping hypothesis

For 13 of the 15 case study MWCNTs the HARN hypothesis was rejected due to insufficient length or rigidity, and therefore these MWCNT should not be included in a group based on common fate or hazard potential dictated by fibre morphology. Needle-like morphology is considered a driver of fibre toxicity [8]. Although studies have reported adverse pulmonary outcomes for a number of MWCNT within this panel which do not present a needle-like morphology (Table S4), [12, 13, 15, 46, 47] the mechanism of toxicity leading to the disease may be different. Indeed, this is clearly demonstrated by Gate et al. [15] who directly compared two MWCNT included in this case study which did not fall into the same group. Although needle-like NM-401 and non-needle-like NM-403 both induced some degree of lung pathology after inhalation exposure the underlying transcriptional and proteomic changes differed significantly suggesting the same molecular changes are not triggered by these diverse MWCNT. It would be inappropriate to assume MWCNT operating via divergent toxic pathways will likely induce the same disease endpoints in humans with high levels of scientific confidence. Rejection of a grouping hypothesis does not lead to a conclusion of no hazard but prompts the user to re-enter the Framework and select an alternative relevant pre-defined hypothesis to support the grouping of the remaining MWCNT. If a group can be formed from the remaining 13 MWCNT read-across of data from MWCNT where hazard data exists to group members where it does not, may be possible if sufficient similarity can be demonstrated between group members. Within this case study, we have already addressed the question of dissolution and concluded that the remaining MWCNT can be considered slowly dissolving or effectively biopersistent based on their graphenic structure. An alternative GRACIOUS pre-defined grouping hypothesis which supports grouping NFs with a very slow dissolution rate based on similar reactivity and inflammatory potential could be tested for grouping these MWCNT as ‘respirable NFs with a very slow dissolution rate, which following chronic inhalation exposure will accumulate in the lungs and lead to long-term toxicity’ [6].

Alternative or additional grouping hypotheses may also be needed to consider the broader hazard posed by MWCNT to the respiratory tract. The HARN IATA was developed building on the fibre pathogenicity paradigm which describes the drivers of toxicity for pathogenic fibres such as asbestos. Common adverse outcomes associated with asbestos exposure are reported to arise from the alveolar region of the lung (deposition at the bronchoalveolar bifurcation) and the pleural cavity and therefore more likely driven by the respirable fraction than the inhalable fraction. Furthermore, from a regulatory perspective the recommended exposure limits refer to the respirable and not inhalable fraction (NIOSH Current Bulletin). Mitsui-7 (NRCWE006), the most studied MWCNT fits within this paradigm and as such has driven the comparison of all MWCNT with pathogenic fibres such as asbestos. However, for some MWCNT the propensity to form large agglomerates may lead to greater deposition in the bronchial region, therefore the major target site for toxicity may be higher in the respiratory tract. An alternative grouping hypothesis would need to be generated to assess the similarity of different MWCNT in terms of hazard potential in the bronchiolar region or the upper respiratory tract. Evidence is increasing that the secondary structure i.e. the agglomerated form of the MWCNT may be indicative of hazard potential. More work needs to be done with realistic industrially-relevant forms of MWCNT to determine how best to report this parameter in a standardised manner and how it may be utilised in grouping an hazard assessment of non-rigid, tangled, highly agglomerated MWCNT such as the 13 case study MWCNT which were excluded from the group here.

## Implications of the similarity assessment

Pairwise similarity assessment based on expert opinion and analysis of the data matrix judged the similarity between the grouped MWCNT to be sufficient to support building a read-across argument for the interpolation of hazard data from source (NRCWE006) to target (NM-401). The assessment of similarity presented here is restricted to a simple pairwise comparison of a time/dose endpoint (maximum response) or summary metric (e.g. EC_50_) to represent each of the hazard outcomes tested. Alternative approaches for modelling of similarity which utilise the full set of dose–response data could be more representative of the similarity of hazard responses elicited by the MWCNT in the Tier 1 in vitro assays as the shape and magnitude of the dose–response curves could also be taken into account, allowing temporal differences or differences in potency to be recognised [14]. Similarity results from more complete data sets could reduce uncertainty and add confidence to the interpretation of similarity of hazard potential.

Acceptance of the argument that NM-401 is similar to NRCWE006 could be used to fill hazard data gaps that would have significant implications for the regulation of NM-401 and other MWCNT shown to group with these MWCNT with a high degree of similarity in future. For NM-401 hazard data generated according to OECD TG 412 (28-day study, sub-acute inhalation) exists but no longer-term hazard data from sub-chronic or chronic studies are available. Recently results from both a 13-week sub-chronic inhalation study, following OECD TG 413 [18] and a long-term 2-year carcinogenicity study using whole-body inhalation exposure (OECD TG 435, [19]) have been reported for NRCWE006 (Mitsui-7) MWCNT using male and female F344 rats. Granulomatous changes in the tissue and a persistent inflammation was noted after 13-weeks exposure to high concentrations of NRCWE006 (1 and 5 mg/m^3^). After 104-weeks exposure, concentration-dependent toxic effects in the lung such as epithelial hyperplasia, granulomatous change, localized fibrosis, and alteration in lavage fluid parameters were reported in exposure groups of both sexes. Furthermore, lung carcinomas were significantly increased in both male and female rats exposed to the highest concentration of 2 mg/m^3^ (16/100 rats). If similarity between NRCWE006 and NM-401 was accepted by regulators, the results of the Kasai sub-chronic study could be used to fill data gaps for NM-401 based on the assumption that long-term sub-chronic inhalation exposure of NM-401 will cause pathological changes with a similar potency. The implication of this outcome is the prediction that NM-401 is similarly carcinogenic, an adverse outcome not yet demonstrated in vivo after exposure to NM-401. In the short-term inhalation study conducted with NM-401 an acute inflammatory response appeared to regress after termination of inhalation exposure [15]. However changes in the transcriptional and translational expression of genes and proteins in key pathways involved in chronic disease such as fibrosis persisted in the post-exposure period, suggesting the potential for pathogenesis of long-term disease upon continued exposure to NM-401 [39].

Assessment of mesothelioma formation in response to inhaled substances is not specifically covered by the OECD TG and as such is not a hazard endpoint required by regulatory agencies, however it remains a major concern given the burden of disease linked with asbestos exposure. Due to technical limitations of rodent models, this endpoint may not be detected when conducting inhalation studies due to the long latency of human disease, therefore a negative result in such models cannot be confidently interpreted as a lack of mesothelioma hazard. Although limited in physiological relevance for risk assessment, alternative routes of exposure have been used to indicate the potential to pose a mesothelioma hazard based on direct injection of material into the pleural or peritoneal cavities. NRCWE006 has been shown, in multiple studies, to induce mesothelioma formation in mice and rats after direct injection into the peritoneal and intrascrotal cavity [23, 25, 48–50] and intra-tracheal intra-pulmonary spraying [51, 52]. To date no studies have tested the carcinogenicity of NM-401 in models of mesothelioma formation. Acceptance that the degree of similarity between NRCWE006 and NM-401 presented indicates a similar hazard potential would suggest the carcinogenicity of NM-401 leading to mesothelioma formation could be predicted from existing NRCWE006 hazard data. In this instance read-across of carcinogenicity in both the lung and mesothelium could support the adoption of the IARC classification for Mitsui-7/NRCWE006 as a Group 2B (possible human carcinogen) for NM-401 with an acceptable degree of confidence without the need for further in vivo studies to be conducted. Importantly the lack of similarity between NRCWE006 and the 2 dissimilar MWCNT for which the grouping hypothesis was rejected (NM-403 and NRCWE040) demonstrates how the grouping hypothesis and IATA presented here may be used to support a nuanced and evidence-based regulation of MWCNT without the need to provide extensive in vivo evidence to differentiate between the hazard posed by different NFs.

## Refinement of the IATA

Practical application of this case study has allowed the performance of the HARN IATA originally presented in [7] to be tested and the potential need for refinement to be assessed. The appropriateness and essentiality of each decision node to support grouping was considered. Frustrated phagocytosis is a direct outcome of the inability of macrophages to completely engulf and passivate high aspect ratio materials leading to lysosomal disruption and the activation of NALP3 inflammasome resulting in the release of the pro-inflammatory cytokine, IL-1β [28]. The decision node specifically addressing the ability of HARN to elicit frustrated phagocytosis was included with the aim of strengthening the mechanistic underpinning of a group. However, as a measurable biological endpoint it is difficult to define and no standard method to assess frustrated phagocytosis yet exists. In the IATA IL-1β release and CathepsinB release and evidence of lysosomal disruption after incubation of macrophages with HARN have been selected as simple endpoints indicative of frustrated phagocytosis [7]. However, these endpoints could also be induced by alternative modes of action unrelated to fibre morphology such as surface reactivity [53, 54]. Indeed, here we report the release of IL-1β after exposure of THP-1 cells to a number of the MWCNT case study panel which were not considered to cause frustrated phagocytosis based on their physical dimensions and qualitative images showing successful uptake. Therefore, a positive outcome for the frustrated phagocytosis decision node is not considered sufficiently specific to differentiate between HARN which may induce toxic responses via different mechanisms. As a refinement to the IATA it is suggested this decision node should be removed but the endpoints included within the wider hazard assessment alongside multiple hazard endpoints (including reactivity, inflammation, genotoxicity) to support the mechanistic underpinning of the group and similarity assessment to justify read-across between source and target group members.

The argument presented here for the interpolation of hazard data from NRCWE006 to NM-401 is based on the demonstrated similarity in Tier 1 IATA endpoints, however confidence in supporting this argument is largely derived from existing evidence of MWCNT deposition, biopersistence and similarity in hazard outcomes between NRCWE006 and NM-401 from in vivo studies. There is often reluctance to accept read-across arguments based on simple acute in vitro assays as they are considered to have low predictivity for long-term in vivo responses [55], highlighting the need for more physiologically-relevant in vitro models. Cell and tissue-culture models which incorporate multiple cell types and can be subject to repeat or long-term exposures may better replicate the potential pro-inflammatory milieu that promotes the development of adverse histopathological outcomes seen in vivo. Future refinement of the IATA should focus on the inclusion of more sophisticated Tier 2 models to improve the predictivity of IATA grouping and similarity assessment for in vivo hazard outcomes, which will build confidence in the applicability of in vitro data for supporting read-across and reduce the reliance on in vivo testing in future.

## Conclusion

The GRACIOUS approach to support hypothesis-driven grouping utilises IATAs to guide users for the efficient collection of relevant information for grouping, through integration of existing data with new data sets obtained by following a tiered testing strategy if new data is required. The IATAs and tiered testing strategies contained within them were developed from a comprehensive review of the literature to identify measurable drivers of hazard. The case study reports the successful application of the GRACIOUS Framework and HARN IATA to support a hypothesis-based grouping of MWCNT with a predicted similar hazard potential after inhalation exposure. Furthermore, the IATA formed the basis of a structured similarity assessment to build a read-across argument for the interpolation of hazard data between the grouped MWCNT. This proof-of-concept case study demonstrates the use of the GRACIOUS IATA for the streamlined hazard assessment of MWCNT fit for regulatory use and highlights potential refinements to improve the IATA process and facilitate it’s continued use in future.

## Supplementary Information

**Additional file 1: Table S1:** Endpoints included in the NRCWE hazard assessment studies of the MWCNT panel (A). Specific MWCNT included in each study (B).

**Additional file 2: Table S2:** Tier 1 information extracted from the NRCWE studies for each of the MWCNT panel according to the IATA decision nodes.*Assumed density: 1.7ρ_g_ adopted from (Kim et al., 2009).

**Additional file 3: Table S3:** Information gathered from targeted literature search for NM-401 and NRCWE006 to fill data gaps required to progress through the IATA decision nodes.

**Additional file 4: Table S4: **Summary of inhalation studies assessing the hazard of NRCWE006, NM-401, and NM-403 MWCNT.

**Additional file 5: Fig. S1: **NRCWE006, NM-401, NRCWE040 dispersed by ultrasonication in 0.5% BSA/RPMI cell culture media prior to exposure to cells. NRCWE006, NM-401, NRCWE040 and THP-1 cells 24 hours after exposure.

**Additional file 6: Fig. S2:** Scanning microscopy images of MWCNT panel adapted from (Jackson et al., 2015). Jackson, P., Kling, K., Jensen, K. A., Clausen, P. A., Madsen, A. M., Wallin, H., & Vogel, U. (2015). Characterization of genotoxic response to 15 multiwalled carbon nanotubes with variable physicochemical properties including surface functionalizations in the FE1-Muta(TM) mouse lung epithelial cell line. Environmental and Molecular Mutagenesis, 56(2), 183–203.

**Additional file 7: Table S5:** Fold change over no particle control, DCFH Fluorescent arbitrary units. Table S6: IL-1β release from THP-1 cells exposure to MWCNT for 24 hours measured in supernantant by ELISA (R&D systems Duo Set). Exposure co ncentration range 1.56-100µg/ml.

**Additional file 8: Table S6:** Residual metal content for MWCNT case study panel. Data adapted from Jackson et al 2015.

**Additional file 9: Fig. S3:** Transmission Electron microscope images of MWCNT panel. Scale bar = 100µm. MWCNT were dispersed in 70% ethanol and dispersed by ultrasonication prior to mounting on TEM grids.

**Additional file10: **Fig. S4: Light microscopy images of THP-1 cells exposed to MWCNT panel (10µg/ml) for 6 hours. Scale bar = 20µm.

## Data Availability

The dataset(s) supporting the conclusions of this article is(are) included within the article (and its additional file(s)).

## Abbreviations

AOP: Adverse outcome pathway
AR: Aspect ratio
BAL: Bronchial-alveolar lavage
BET: Brunauer–Emmett–Teller
D_ae_: Aerodynamic diameter
DCFH_2_-DA: 2′,7′-Dichlorodihydrofluorescein diacetate
HARN: High aspect ratio nanomaterials
IATA: Integrated approaches to testing and assessment
IARC: International agency for research on cancer
IT: Intratracheal instillation
LDH: Lactate dehydrogenase
MMAD: Mass media aerodynamic diameter
MWCNT: Multiwalled carbon nanotubes
NF: Nanoforms
NM: Nanomaterials
NRCWE: National research centre for the working environment
OECD TG: Organisation for economic co-operation and development test guidelines
PC: Physicochemical characteristics
PMA: Phorbol 12-myristate 13-acetate
QSAR: Quantitative structure activity relationship
ROS: Reactive oxygen species
RTM: Representative test material
SEM: Scanning electron microscopy
STOT-RE: Specific target organ toxicity—repeat exposure
TEM: Transmission electron microscopy
