# Supplementary material for: Grouping MWCNTs based on their similar potential to cause pulmonary hazard after inhalation: a case-study
Source: Part Fibre Toxicol. 2022 Jul 20;19:50. doi: 10.1186/s12989-022-00487-6 (PMC9297605; doi:10.1186/s12989-022-00487-6)
Supplement: Supplementary file 1 — Additional file 1: Table S1: Endpoints included in the NRCWE hazard assessment studies of the MWCNT panel (A). Specific MWCNT included in each study (B). [file 12989_2022_487_MOESM1_ESM.docx]

Additional File 1

Table S1: Endpoints included in the NRCWE hazard assessment studies of the MWCNT panel (A). Specific MWCNT included in each study (B).

| A | (Jackson et al., 2015) | (Poulsen et al., 2016) | (Poulsen et al., 2017) | (Knudsen et al., 2019) | (Di Ianni et al., 2021) |
| --- | --- | --- | --- | --- | --- |
|  | **PC characterization:**  Purity, SEM size measurements, Surface area    **Reactivity:**  DCFH_2_-DA    ***In vitro* hazard:**  FE1MutaTMMouse Lung Epithelial Cell line.  Cytotoxicty and Genotoxicity (Comet assay) after 24 h exposure. | ***In vivo* hazard:**  Intratracheal instillation of 0, 6, 18 or 54 µg/mouse.  Pulmonary inflammation (neutrophil influx in bronchoalveolar lavage (BAL)) and genotoxicity were determined on day 1, 28 or 92. Histopathology of the lungs was performed on day 28 and 92. | ***In vivo* hazard:**  Intratracheal instillation of 0, 6, 18 or 54 µg/mouse.  Plasma levels of acute phase response proteins serum amyloid A1/2 (SAA1/2) and SAA3 were determined on day 1, 28 or 92. Expression levels of hepatic Saa1 and pulmonary Saa3 mRNA levels were assessed to determine the origin of the acute phase response proteins. | ***In vivo* hazard:**  Intratracheal instillation of 54 µg/mouse.    Histological changes in lung tissue at 1 year. Genotoxicity in liver and spleen was evaluated by the Comet assay. | ***In vitro* hazard:**  Human alveolar epithelial cells (A549) and monocyte-derived macrophages (THP-1a) were exposed to in submerged conditions, and a subset of NM in co-cultures of  A549, THP-1a and lung fibroblasts (WI-38) in an air-liquid interface (ALI) system.  Effective doses at 24h were quantified by thermo-gravimetric analysis (TGA).  Inflammation (IL-8 expression) and genotoxicity (Comet assay) were assessed. |

| B | Jackson *et al* 2015 | NM-400 | NM-401 | NM-402 | NM-403 | NRCWE006 | NRCWE040 | NRCWE041 | NRCWE042 | NRCWE043 | NRCWE044 | NRCWE045 | NRCWE046 | NRCWE047 | NRCWE048 | NRCWE049 |
| --- | --- | --- | --- | --- | --- | --- | --- | --- | --- | --- | --- | --- | --- | --- | --- | --- |
|  | Poulsen *et al* 2016 |  |  |  |  |  | NRCWE-040 | NRCWE-041 | NRCWE-042 | NRCWE-043 | NRCWE-044 | NRCWE-045 | NRCWE-046 | NRCWE-047 | NRCWE-048 | NRCWE-049 |
|  | Poulsen *et al* 2017 | NM-400 | NM-401 | NM-402 | NM-403 |  | NRCWE-040 | NRCWE-041 | NRCWE-042 | NRCWE-043 | NRCWE-044 | NRCWE-045 | NRCWE-046 | NRCWE-047 | NRCWE-048 | NRCWE-049 |
|  | Knudsen *et al* 2019 | NM-400 | NM-401 | NM-402 | NM-403 | NRCWE-006 | NRCWE-040 | NRCWE-041 | NRCWE-042 |  |  |  | NRCWE-046 | NRCWE-047 | NRCWE-048 |  |
|  | di Ianni *et al* 2021 | NM-400 | NM-401 |  | NM-403 | NRCWE-006 | NRCWE-040 | NRCWE-041 |  |  |  |  |  |  |  |  |
