# Supplementary material for: Grouping MWCNTs based on their similar potential to cause pulmonary hazard after inhalation: a case-study
Source: Part Fibre Toxicol. 2022 Jul 20;19:50. doi: 10.1186/s12989-022-00487-6 (PMC9297605; doi:10.1186/s12989-022-00487-6)
Supplement: Supplementary file 2 — Additional file 2: Table S2: Tier 1 information extracted from the NRCWE studies for each of the MWCNT panel according to the IATA decision nodes.*Assumed density: 1.7ρg adopted from (Kim et al., 2009). [file 12989_2022_487_MOESM2_ESM.docx]

Additional File 2

Table S2: Tier 1 information extracted from the NRCWE studies for each of the MWCNT panel according to the IATA decision nodes. *Assumed density: 1.7ρ_g_ adopted from (Kim et al., 2009)

|  | **Grouping Decision Nodes**  **Indicative of common fate or hazard potential** | | | | | |
| --- | --- | --- | --- | --- | --- | --- |
| **Tier 1** | **Can NF deposit in the distal lung?** | **Does the NF dissolve very slowly in lung lining fluid?** | **Does the NF dissolve very slowly in lysosomal fluid?** | **Is HARN length >5µm?** | **Is the HARN rigid and maintain a fibrous, needle-like morphology?** | **Does the NF cause frustrated phagocytosis?** |
| **Assay** | **Estimation of aerodynamic diameter (D_ae_) from NF size measurements by TEM/SEM and density measurement.** | **Batch dissolution or continuous flow dissolution in LLF (pH 7.4)** | **Batch dissolution or continuous flow dissolution in lysosomal fluid (pH 4.5)** | **Size measurements by TEM/SEM** | **Size measurements by TEM/SEM** | **Inflammasome activation:**   - **IL-1β release,** - **CathepsinB activity/release** |
| **Criteria** | **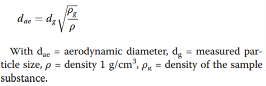**  **D_ae_ > 4µm** | **Half-life < 60days** | **Half-life < 60days** | **Greater than 10% > 5µm length** | **Median diameter > 30nm**  **Supported by EM images** | **Statistically significant, dose-dependent increase over negative control** |
| NM-400 | Mean measured diameter: 11nm  *Assumed density: 1.7ρ_g_  **Estimated D_ae_: 14.34 nm** | **No data available.**  Predicted to be durability based on graphene structure. | **No data available.**  Predicted to be durability based on graphene structure. | **Length distribution not provided**  Mean length:  0.85±0.10 µm | **Mean diameter:**  **11±3 nm**  Supporting SEM provided | **No data available.** |
| NM-401 | Mean measured diameter: 67nm  *Assumed density: 1.7ρ_g_  **Estimated D_ae_: 87.36 nm** | **No data available.**  Predicted to be durability based on graphene structure. | **No data available.**  Predicted to be durability based on graphene structure. | **Length distribution not provided**  Mean length:  4.0±0.37 µm | **Mean diameter:**  **67±24 nm**  Supporting SEM provided | **No data available.** |
| NM-402 | Mean measured diameter: 11nm  *Assumed density: 1.7ρ_g_  **Estimated D_ae_: 14.34 nm** | **No data available.**  Predicted to be durability based on graphene structure. | **No data available.**  Predicted to be durability based on graphene structure. | **Length distribution not provided**  Mean length:  1.4±0.19 µm | **Mean diameter:**  **11±3 nm**  Supporting SEM provided | **No data available.** |
| NM-403 | Mean measured diameter: 12nm  *Assumed density: 1.7ρ_g_  **Estimated D_ae_: 15.65 nm** | **No data available.**  Predicted to be durability based on graphene structure. | **No data available.**  Predicted to be durability based on graphene structure. | **Length distribution not provided**  Mean length:  0.4±0.03 µm | **Mean diameter:**  **12±7 nm**  Supporting SEM provided | **No data available.** |
| NRCWE-006 | Mean measured diameter: 74nmn  *Assumed density: 1.7ρ_g_  **Estimated D_ae_: 96.48 nm** | **No data available.**  Predicted to be durability based on graphene structure. | **No data available.**  Predicted to be durability based on graphene structure. | **Length distribution not provided**  Mean length:  5.7±0.49 µm | **Mean diameter:**  **74 (29-173) nm**  Supporting SEM provided | **No data available.** |
| NRCWE040 | Mean measured diameter: 20.56nm  *Assumed density: 1.7ρ_g_  **Estimated D_ae_: 26.80 nm** | **No data available.**  Predicted to be durability based on graphene structure. | **No data available.**  Predicted to be durability based on graphene structure. | **Length distribution not provided**  Mean length:  0.52±0.59 µm | **Mean diameter:**  **20.56±6.9 nm**  Supporting SEM provided | **No data available.** |
| NRCWE041 | Mean measured diameter: 26.38nm  *Assumed density: 1.7ρ_g_  **Estimated D_ae_: 34.4 nm** | **No data available.**  Predicted to be durability based on graphene structure. | **No data available.**  Predicted to be durability based on graphene structure. | **Length distribution not provided**  Mean length:  1.0±2.95 µm | **Mean diameter:**  **26.38±11.1 nm**  Supporting SEM provided | **No data available.** |
| NRCWE-042 | Mean measured diameter: 20.5nmn  *Assumed density: 1.7ρ_g_  **Estimated D_ae_: 26.73 nm** | **No data available.**  Predicted to be durability based on graphene structure. | **No data available.**  Predicted to be durability based on graphene structure. | **Length distribution not provided**  Mean length:  0.72±0.97 µm | **Mean diameter:**  **20.5±5.32 nm**  Supporting SEM provided | **No data available.** |
| NRCWE-043 | Mean measured diameter: 26.73nm  *Assumed density: 1.7ρ_g_  **Estimated D_ae_: 34.85nm** | **No data available.**  Predicted to be durability based on graphene structure. | **No data available.**  Predicted to be durability based on graphene structure. | **Length distribution not provided**  Mean length:  0.771±3.47 µm | **Mean diameter:**  **26.73±6.88 nm**  Supporting SEM provided | **No data available.** |
| NRCWE-044 | Mean measured diameter: 32.55nm  *Assumed density: 1.7ρ_g_  **Estimated D_ae_: 42.44nm** | **No data available.**  Predicted to be durability based on graphene structure. | **No data available.**  Predicted to be durability based on graphene structure. | **Length distribution not provided**  Mean length:  1.33±2.454 µm | **Mean diameter:**  **32.55±14.4 nm**  Supporting SEM provided | **No data available.** |
| NRCWE-045 | Mean measured diameter: 28.07nm  *Assumed density: 1.7ρ_g_  **Estimated D_ae_: 36.6nm** | **No data available.**  Predicted to be durability based on graphene structure. | **No data available.**  Predicted to be durability based on graphene structure. | **Length distribution not provided**  Mean length:  1.553±2.954 µm | **Mean diameter:**  **28.07±13.55 nm**  Supporting SEM provided | **No data available.** |
| NRCWE-046 | Mean measured diameter: 17.2nm  *Assumed density: 1.7ρ_g_  **Estimated D_ae_: 22.43 nm** | **No data available.**  Predicted to be durability based on graphene structure.. | **No data available.**  Predicted to be durability based on graphene structure. | **Length distribution not provided**  Mean length:  0.72±1.2 µm | **Mean diameter:**  **17.2±5.8 nm**  Supporting SEM provided | **No data available.** |
| NRCWE-047 | Mean measured diameter: 12.96nmn  *Assumed density: 1.7ρ_g_  **Estimated D_ae_: 16.9 nm** | **No data available.**  Predicted to be durability based on graphene structure. | **No data available.**  Predicted to be durability based on graphene structure. | **Length distribution not provided**  Mean length:  0.53±0.59 µm | **Mean diameter:**  **12.96±4.4 nm**  Supporting SEM provided | **No data available.** |
| NRCWE-048 | Mean measured diameter: 15.08nm  *Assumed density: 1.7ρ_g_  **Estimated D_ae_: 19.66 nm** | **No data available.**  Predicted to be durability based on graphene structure. | **No data available.**  Predicted to be durability based on graphene structure. | **Length distribution not provided**  Mean length:  1.6±5.6 µm | **Mean diameter:**  **15.08±4.7 nm**  Supporting SEM provided | **No data available.** |
| NRCWE-049 | Mean measured diameter: 13.85nm  *Assumed density: 1.7ρ_g_  **Estimated D_ae_: 18.05 nm** | **No data available.**  Predicted to be durability based on graphene structure. | **No data available.**  Predicted to be durability based on graphene structure. | **Length distribution not provided**  Mean length:  0.731±1.473 µm | **Mean diameter:**  **13.85±6.09 nm**  Supporting SEM provided | **No data available.** |
