# Supplementary material for: Grouping MWCNTs based on their similar potential to cause pulmonary hazard after inhalation: a case-study
Source: Part Fibre Toxicol. 2022 Jul 20;19:50. doi: 10.1186/s12989-022-00487-6 (PMC9297605; doi:10.1186/s12989-022-00487-6)
Supplement: Supplementary file 3 — Additional file 3: Table S3: Information gathered from targeted literature search for NM-401 and NRCWE006 to fill data gaps required to progress through the IATA decision nodes. [file 12989_2022_487_MOESM3_ESM.docx]

Additional File 3

Table S3: Information gathered from targeted literature search for NM-401 and NRCWE006 to fill data gaps required to progress through the IATA decision nodes.

| **Decision Node 1:**  **Can NF deposit in the distal lung?** | | |
| --- | --- | --- |
| **Tier 1** | | |
|  | **Assay: Estimation of aerodynamic diameter (D_ae_) from NF size measurements by TEM/SEM and density measurement.** | |
| **MWCNT** | **Criteria : D_ae_ < 4µm** | **DN Outcome** |
| NM-401 | Estimation of D_ae_ not appropriate for heterogenous MWCNT (F. Murphy et al., 2021) | Escalate to Tier 2 |
| NRCWE006 | Estimation of D_ae_ not appropriate for heterogenous MWCNT (F. Murphy et al., 2021) | Escalate to Tier 2 |
| **Tier 2** | | |
|  | **Assay: Measure MMAD by cascade impactor from an airborne dispersion of the material** | |
| **MWCNT** | **Criteria: MMAD < 4µm** | **DN Outcome** |
| NM-401 | 0.79 µm (GSD 1.83) (Gaté et al., 2019)  1.5 µm (GSD 1.67) (Porter et al., 2013) | Yes |
| NRCWE006 (Mitsui-7) | 1.2-1.4 µm (GSD 2.6-3)  Range across exposure groups and duration  (Kasai et al., 2016) | Yes |
| **Tier 3** | | |
| **Assay** | **Quantification of lung burden after in vivo inhalation studies. Both short-term or long-term protocols are considered appropriate.** | |
|  | **Criteria: Evidence of HARN deposition in the distal lung/Evidence of HARN translocation to the pleural cavity.** | **DN Outcome** |
| NM-401 | (Gaté et al., 2019) estimated that 709 and 279 μg of NM-401 were deposited within the rats' thoracic region (tracheobronchial and pulmonary areas) after one month of inhalation at 1.5 and 0.5 mg/m^3^, respectively. One third of the deposited mass reached the pulmonary region. | Yes |
| NRCWE006 | (Kasai et al., 2016) reported the MWNT-7 (NRCWE006) deposited in the lungs of male rats exposed to 0.02, 0.2, and 2 mg/m^3^ MWNT-7 resulted in total lung burdens of approximately 0.01, 0.15, and 1.8 mg.  (Kasai et al., 2016) reported 1 × 10^3^ fibers in the pleura of the 2 mg/m^3^ MWNT-7 exposed animals.  (Mercer et al., 2013): Mice were exposed to a MWCNT aerosol (5mg/m^3^, 5 hours/day) for 12 days. At day 1 post-inhalation exposure 84 ± 3 and 16 ± 2 percent of the lung burden (Mean ± S.E., N = 5) were in the alveolar and airway regions, respectively. | Yes |
|  | | |
| **Decision Node 2:**  **Does the NF dissolve very slowly in lung lining fluid?**  **Does the NF dissolve very slowly in lysosomal fluid?** | | |
| **Tier 1** | | |
| **Assay** | **Batch dissolution or continuous flow dissolution in LLF (pH 7.4)**  **Batch dissolution or continuous flow dissolution in lysosomal fluid (pH 4.5)** | |
| **Criteria** | **Half-life < 60days** | **DN Outcome** |
| NM-401 | No data from additional studies identified | Generate data or escalate to Tier 2 or 3 |
| NRCWE006 | (Osmond-McLeod et al., 2011)  Incubation in Gambles pH4.5 for up to 24 weeks. Mitsui-7(NRCWE006) lost 30% of original mass in the first 3 weeks after which there was no further loss. | Generate data or escalate to Tier 2 or 3 |
| **Tier 2** | | |
| **Assay** | **Durability in cellular system** | |
| **Criteria** | **No threshold criteria set- assess similarity between NF** | **DN Outcome** |
| NM-401 | No data from additional studies identified | Escalate to Tier 3 |
| NRCWE006 | No data from additional studies identified | Escalate to Tier 3 |
| **Tier 3** | | |
| **Assay** | **Quantification of lung burden and clearance kinetics after in vivo inhalation studies.** | |
| **Criteria** | **Half-life < 60days** | **DN Outcome** |
| NM-401 | Qualitative evidence of biopersistence. NM-401 observed intracellularly and in tissue over extended recovery period up to 180 days post exposure. The enhanced darkfield microscopy revealed that in addition to mainly being phagocytized by alveolar macrophages, single or small bundles of NM-401 were also present in the interstitium after both types of exposure and at all timepoints analyzed (3, 30, 90 and 180 days post-inhalation). (Gaté et al., 2019) | Yes |
| NRCWE006 | (Mercer et al., 2013): Mice were exposed to a MWCNT aerosol (5mg/m^3^, 5 hours/day) for 12 days. At 336 days, 65 percent remained in the lungs.  Further qualitative evidence of biopersistence. Presence of NRCWE006 in lung tissue at one year post IT exposure (Knudsen et al., 2019), 2 years after inhalation exposure (Kasai et al., 2016). | Yes |
|  | | |
| **Decision Node 3:**  **Is HARN length >5µm?** | | |
| **Tier 1** | | |
| **Assay** | **Size measurements by TEM/SEM from aerosolized sample** | |
| **Criteria** | **Greater than 10% > 5µm length** | **DN Outcome** |
| NM-401 | No additional data available | Generate data or escalate to Tier 2 or 3 |
| NRCWE006 | 27.5% > 5µm Dispersed in 5% Triton-X 100. (Takagi et al., 2008)  Median 7.1 µm, 78% fibres > 5µm (Huaux et al., 2016)  Mean: 6.65 µm, 54% fibres > 5µm (Sakamoto et al., 2018) | Yes |
| Tier 2 | | |
| Assay | **Size measurements by TEM/SEM from aerosolized sample** | |
| **Criteria** | **Individual fibres > 5µm**  **Secondary structure that meet the WHO fibre criteria (5µm in length, aspect ratio >3:1)** | **DN Outcome** |
| NM-401 | No size profile available. (Gaté et al., 2019) showed representative TEM of aerosol generated for inhalation study showing predominance of fibres > 5µm. | Yes |
| NRCWE006 (Mitsui-7) | For inhalation study:  (Chen et al., 2012) (MWNT-7, lot 061220-31) separated the MWCNT particles into two categories, fiber-like (fibrous) particles and isometric particles from SEM images of aerosolized MWCNT collected onto filters. Approximately 20–40% of the particles were classified as isometric particles and the remaining 60–80% were classified as fibrous particles. 3–5% of the total particles (or particle structures) counted were individual particles, 35–50% of them contained less than 10 nanotubes, and only 6–10% contained more than 30 nanotubes. The fibrous particle width ranged from 15 nm to greater than 500 nm and particle length varied between 0.2 µm and greater than 15 µm. Fibrous particles within the aerosol had a count median length of 3.04 µm and a width of 100.3 nm.  (Kasai et al., 2014) Size measurements from SEM images of aerosolized MWCNT.  Mean length 5.7µm,  median 4.8µm  Min-max: 0.7-22.9µm  Mean diameter: 130.5nm  Median: 91.8nm  Min-max: 30.2-1389.9nm  (Kasai et al., 2015) (MWNT-7, Lot No. 071223, 080126). Size measurement of the aerosolized MWCNT from MOUDI had a mean width of 96.4, 98.0 and 94.1nm and a mean length of 5.83, 6.19 and 5.53mm, for 0.2, 1 and 5mg/m^3^ groups | Yes |
| **Tier 3** | | |
| **Assay** | **No Tier 3 assay recommended for this Decision Node.** | |
|  | | |
| **Decision Node 4:**  **Is the HARN rigid and maintain a needle-like morphology?** | | |
| **Tier 1** | | |
| **Assay** | **Size measurements by TEM/SEM** | |
| **Criteria** | **Median diameter > 30nm, Supported by EM images** | **DN Outcome** |
| NM401 | **Sufficient data from primary panel**  **No contradictory data identified**  Additional relevant study: (Fortini et al., 2020)  Using individual tube diameters and lengths, flexural rigidity values were determined. The flexural rigidity 𝑅 of a fiber is defined as product of bending modulus 𝐸𝑏 and second moment of axial area 𝐼𝑎 (related to 4th power of their fiber diameter 𝐷). By detecting resonance frequencies of cantilevered MWCNTs and applying the Euler–Bernoulli beam theory, bending modulus values 𝐸𝑏 of individual MWCNTs were derived. Authors concluded that NM-401 ‘presented flexural rigidity in the order of 10−18 N∙m2 and therefore can be classified as rigid and potential agents of frustrated phagocytosis’. | **Yes** |
| NRCWE006 | **Sufficient data from primary panel**  No contradictory data identified | **Yes** |
| **Tier 2** | | |
| **Assay** | **Size measurements by TEM/SEM from aerosolized sample** | |
| **Criteria** | **Individual fibres > 5µm**  **Secondary structure that meet the WHO fibre criteria (length > 5µm and aspect ratio > 3:1)** | **DN Outcome** |
| NM-401 | No quantitative data from additional studies identified  (Gaté et al., 2019) show representative TEM of aerosol generated for inhalation study showing predominance of fibres > 5µm. | Generate data or escalate to Tier 3 |
| NRCWE-006 (Mitsui-7) | For inhalation study:  (Chen et al., 2012) (MWNT-7, lot 061220-31)  Visually separated the MWCNT particles into two categories, fiber-like (fibrous) particles and isometric particles. Approximately 20–40% of the particles were classified as isometric particles and the remaining 60–80% were classified as fibrous particles. 3–5% of the total particles (or particle structures) counted were individual particles, 35–50% of them contained less than 10 nanotubes, and only 6–10% contained more than 30 nanotubes. The fibrous particle width ranged from 15 nm to greater than 500 nm and particle length varied between 0.2 µm and greater than 15 µm. Fibrous particles within the aerosol had a count median length of 3.04 µm and a width of 100.3 nm.  (Kasai et al., 2015) (MWNT-7, Lot No. 071223, 080126). Tier 2 measurement of the MWCNT from MOUDI had a mean width of 96.4, 98.0 and 94.1nm and a mean length of 5.83, 6.19 and 5.53mm, for 0.2, 1 and 5mg/m3 groups, aspect ratio >3 | Yes |
| **Tier 3** | | |
| **Assay** | **Characterization of morphology and fibre size after in vitro/in vivo incubation with macrophages to confirm ‘biological stiffness’.** | |
| **Criteria** | **Evidence of maintenance of fibrous, needle-like morphology and length > 5µm after uptake by cells** | **DN Outcome** |
| NM-401 | (Di Ianni et al., 2021; Gaté et al., 2019; Købler et al., 2015) presented light microscopy images of macrophages from BAL fluid of mice exposure to NM-401 showing presence of long, straight, rigid fibres within cells.  (Gaté et al., 2019) further present enhanced darkfield microscopy images of lung showing straight, rigid NM-401 particles mainly located in alveolar macrophages and are also present as single fibers or bundled fibers at or in alveolar walls. | Yes |
| NRCWE006 | (Di Ianni et al., 2021; Duke et al., 2017) presented light microscopy images of macrophages from BAL fluid of mice exposure to NRCWE006 showing presence of long, straight, rigid fibres within cells. | Yes |
|  | | |
| **Decision Node 5:**  **Does the HARN cause frustrated phagocytosis?** | | |
| **Tier 1** | | |
| **Assay** | **Inflammasome activation:**   - **IL-1β release,** - **CathepsinB activity/release** | |
| **Criteria** | **No threshold criteria set- assess similarity between NF** | **DN Outcome** |
| NM401 | No data from additional studies identified | **Generate data or escalate to Tier 2 or 3** |
| NRCWE006 | (Hindman & Ma, 2019) J774A.1 treated with 2.5µg/ml CNT for 24 hour dispersed in culture media with 1% FCS followed by 1 hr stimulation with 5mM ATP. Significant IL-1β mRNA expression and IL-1β secretion. NRLP3 activation confirmed by ASC-Speck formation, caspase1 cleavage and activity.  (Palomäki et al., 2011)  LPS-primed primary macrophages, IL-1β secretion 6 hr exposure. Secretion of IL-18. NRLP3 siRNA inhibited IL-1β secretion. NRLP3 activation was dependent on ROS and CathepsinB.  (Palomäki et al., 2015)  Secretomic analysis of macrophage response. Human MDM exposure for 6 hrs, secreted proteins analysed by 2D-DIGE ad LC-MS/MS. Secretion of lysosomal proteins and proteins linked with inflammation and apoptosis.  (Lee et al., 2018)  Incubated with THP-1 cells 12.5, 25, 50µg/ml for 24 hours. 50µg/ml induced significant loss of viability by MTS. Significant dose-dependent increase in IL-1β, IL-6 and TNFα.  (Boyles et al., 2015)  J774A.1 macrophages no significant increase in IL-1β secretion at 4, 24 or 48hr for dose response7.5-125µg/ml. Visualisation of frustrated phagocytosis by SEM reported.  (F. A. Murphy et al., 2012)  IL-1β release from THP-1 macrophages after 24 hour incubation. Dose: 5µg/cm2. Significant increase in IL-1β. Blocking phagocytosis with cytochalasin D inhibited increase in IL-1β.  (Cui et al., 2014)  Differentiated THP-1 cells treated for 6 hours, viability assessed by LDH. Dose response 0-20µg/ml. Significant increase in IL-1β in supernatant from dose 6.6µg/ml. IL-1β release inhibited by cytochalsinD. Increase in active Caspase1 cleavage fragment observed by wester blotting. IL-1β secretion inhibited by capase1 inhibitor zYVAD-fmk and excess KCL in medium. IL-1β secretion reduced by NLRP3 siRNA.  (Chortarea et al., 2018)  24 hour exposure of THP-1 cells to NRCWE006 caused a significant increase in IL-1β release.  (Zhu et al., 2016)  Incubation with hepatocytes for 24hrs. CathepsinB release with both 10µg/ml and 20µg/ml dose. Lysosomal membrane permeabilization | **Yes** |
| **Tier 2** | | |
| **Assay** | **In vitro granuloma formation** | |
| **Criteria** | **Formation of stable granuloma after 10 culture in soft agar** | **DN Outcome** |
| NM-401 | No data from additional studies identified | Generate data or escalate to Tier 3 |
| NRCWE006 (Mitsui-7) | (Sanchez et al., 2011)  Primary murine BMDM exposed to MWCNT induced macrophage differentiation into epithelioid cells and formation of stable aggregates with characteristic morphology of granulomas and formation of multinucleated giant cells. Co-expressed M1 and M2 phenotypic markers. | Yes |
| **Tier 3** | | |
| **Assay** | **Characterization of morphology and fibre size after in vitro/in vivo incubation with macrophages.** | |
| **Criteria** | **Qualitative evidence of frustrated phagocytosis** | **DN Outcome** |
| NM-401 | (Købler et al., 2015)studied the ultra-structural time course of CNT distribution in vivo 1, 3 and 28 days after intratracheal instillation of CNTs in mice with a focus on TEM imaging. NM-401 caused clear deformation of the vesicles inside alveolar macrophages, and appeared to be involved in the process of vesicular escape (‘vesicular escape’) which may equate to lysosomal disruption. | Yes |
| NRCWE-006 | (Zhu et al., 2016) showed lysosomal disruption within macrophages exposure to NRCWE006 in vitro leading to inflammasome activation and IL-1β release. | Yes |

Boyles, M. S. P., Young, L., Brown, D. M., MacCalman, L., Cowie, H., Moisala, A., Smail, F., Smith, P. J. W., Proudfoot, L., Windle, A. H., & Stone, V. (2015). Multi-walled carbon nanotube induced frustrated phagocytosis, cytotoxicity and pro-inflammatory conditions in macrophages are length dependent and greater than that of asbestos. Toxicology in Vitro, 29(7), 1513–1528. https://doi.org/https://doi.org/10.1016/j.tiv.2015.06.012

Chen, B. T., Schwegler-Berry, D., McKinney, W., Stone, S., Cumpston, J. L., Friend, S., Porter, D. W., Castranova, V., & Frazer, D. G. (2012). Multi-walled carbon nanotubes: sampling criteria and aerosol characterization. Inhalation Toxicology, 24(12), 798–820. https://doi.org/10.3109/08958378.2012.720741

Chortarea, S., Zerimariam, F., Barosova, H., Septiadi, D., Clift, M. J. D., Petri-Fink, A., & Rothen-Rutishauser, B. (2018). Profibrotic Activity of Multiwalled Carbon Nanotubes Upon Prolonged Exposures in Different Human Lung Cell Types. Applied In Vitro Toxicology, 5(1), 47–61. https://doi.org/10.1089/aivt.2017.0033

Cui, H., Wu, W., Okuhira, K., Miyazawa, K., Hattori, T., Sai, K., Naito, M., Suzuki, K., Nishimura, T., Sakamoto, Y., Ogata, A., Maeno, T., Inomata, A., Nakae, D., Hirose, A., & Nishimaki-Mogami, T. (2014). High-temperature calcined fullerene nanowhiskers as well as long needle-like multi-wall carbon nanotubes have abilities to induce NLRP3-mediated IL-1β secretion. Biochemical and Biophysical Research Communications, 452(3), 593–599. https://doi.org/https://doi.org/10.1016/j.bbrc.2014.08.118

Di Ianni, E., Erdem, J. S., Møller, P., Sahlgren, N. M., Poulsen, S. S., Knudsen, K. B., Zienolddiny, S., Saber, A. T., Wallin, H., Vogel, U., & Jacobsen, N. R. (2021). In vitro-in vivo correlations of pulmonary inflammogenicity and genotoxicity of MWCNT. Particle and Fibre Toxicology, 18(1), 25. https://doi.org/10.1186/s12989-021-00413-2

Duke, K. S., Taylor-Just, A. J., Ihrie, M. D., Shipkowski, K. A., Thompson, E. A., Dandley, E. C., Parsons, G. N., & Bonner, J. C. (2017). STAT1-dependent and -independent pulmonary allergic and fibrogenic responses in mice after exposure to tangled versus rod-like multi-walled carbon nanotubes. Particle and Fibre Toxicology, 14(1), 26. https://doi.org/10.1186/s12989-017-0207-3

Fortini, R., Meyer-Plath, A., Kehren, D., Gernert, U., Jácome, L. A., & Sturm, H. (2020). Measurement of Flexural Rigidity of Multi-Walled Carbon Nanotubes by Dynamic Scanning Electron Microscopy. In Fibers (Vol. 8, Issue 5). https://doi.org/10.3390/fib8050031

Gaté, L., Knudsen, K. B., Seidel, C., Berthing, T., Chézeau, L., Jacobsen, N. R., Valentino, S., Wallin, H., Bau, S., Wolff, H., Sébillaud, S., Lorcin, M., Grossmann, S., Viton, S., Nunge, H., Darne, C., Vogel, U., & Cosnier, F. (2019). Pulmonary toxicity of two different multi-walled carbon nanotubes in rat: Comparison between intratracheal instillation and inhalation exposure. Toxicology and Applied Pharmacology, 375, 17–31. https://doi.org/https://doi.org/10.1016/j.taap.2019.05.001

Hindman, B., & Ma, Q. (2019). Carbon nanotubes and crystalline silica stimulate robust ROS production, inflammasome activation, and IL-1β secretion in macrophages to induce myofibroblast transformation. Archives of Toxicology, 93(4), 887–907. https://doi.org/10.1007/s00204-019-02411-y

Huaux, F., d’Ursel de Bousies, V., Parent, M.-A., Orsi, M., Uwambayinema, F., Devosse, R., Ibouraadaten, S., Yakoub, Y., Panin, N., Palmai-Pallag, M., van der Bruggen, P., Bailly, C., Marega, R., Marbaix, E., & Lison, D. (2016). Mesothelioma response to carbon nanotubes is associated with an early and selective accumulation of immunosuppressive monocytic cells. Particle and Fibre Toxicology, 13(1), 46. https://doi.org/10.1186/s12989-016-0158-0

Jackson, P., Kling, K., Jensen, K. A., Clausen, P. A., Madsen, A. M., Wallin, H., & Vogel, U. (2015). Characterization of genotoxic response to 15 multiwalled carbon nanotubes with variable physicochemical properties including surface functionalizations in the FE1-Muta(TM) mouse lung epithelial cell line. Environmental and Molecular Mutagenesis, 56(2), 183–203. https://doi.org/https://doi.org/10.1002/em.21922

Kasai, T., Gotoh, K., Nishizawa, T., Sasaki, T., Katagiri, T., Umeda, Y., Toya, T., & Fukushima, S. (2014). Development of a new multi-walled carbon nanotube (MWCNT) aerosol generation and exposure system and confirmation of suitability for conducting a single-exposure inhalation study of MWCNT in rats. Nanotoxicology, 8(2), 169–178. https://doi.org/10.3109/17435390.2013.766277

Kasai, T., Umeda, Y., Ohnishi, M., Kondo, H., Takeuchi, T., Aiso, S., Nishizawa, T., Matsumoto, M., & Fukushima, S. (2015). Thirteen-week study of toxicity of fiber-like multi-walled carbon nanotubes with whole-body inhalation exposure in rats. Nanotoxicology, 9(4), 413–422. https://doi.org/10.3109/17435390.2014.933903

Kasai, T., Umeda, Y., Ohnishi, M., Mine, T., Kondo, H., Takeuchi, T., Matsumoto, M., & Fukushima, S. (2016). Lung carcinogenicity of inhaled multi-walled carbon nanotube in rats. Particle and Fibre Toxicology, 13(1), 53. https://doi.org/10.1186/s12989-016-0164-2

Kim, S. H., Mulholland, G. W., & Zachariah, M. R. (2009). Density measurement of size selected multiwalled carbon nanotubes by mobility-mass characterization. Carbon, 47(5), 1297–1302. https://doi.org/https://doi.org/10.1016/j.carbon.2009.01.011

Knudsen, K. B., Berthing, T., Jackson, P., Poulsen, S. S., Mortensen, A., Jacobsen, N. R., Skaug, V., Szarek, J., Hougaard, K. S., Wolff, H., Wallin, H., & Vogel, U. (2019). Physicochemical predictors of Multi-Walled Carbon Nanotube–induced pulmonary histopathology and toxicity one year after pulmonary deposition of 11 different Multi-Walled Carbon Nanotubes in mice. Basic & Clinical Pharmacology & Toxicology, 124(2), 211–227. https://doi.org/10.1111/bcpt.13119

Købler, C., Poulsen, S. S., Saber, A. T., Jacobsen, N. R., Wallin, H., Yauk, C. L., Halappanavar, S., Vogel, U., Qvortrup, K., & Mølhave, K. (2015). Time-dependent subcellular distribution and effects of carbon nanotubes in lungs of mice. PloS One, 10(1), e0116481–e0116481. https://doi.org/10.1371/journal.pone.0116481

Lee, D.-K., Jeon, S., Han, Y., Kim, S.-H., Lee, S., Yu, I. J., Song, K. S., Kang, A., Yun, W. S., Kang, S.-M., Huh, Y. S., & Cho, W.-S. (2018). Threshold Rigidity Values for the Asbestos-like Pathogenicity of High-Aspect-Ratio Carbon Nanotubes in a Mouse Pleural Inflammation Model. ACS Nano, 12(11), 10867–10879. https://doi.org/10.1021/acsnano.8b03604

Mercer, R. R., Scabilloni, J. F., Hubbs, A. F., Battelli, L. A., McKinney, W., Friend, S., Wolfarth, M. G., Andrew, M., Castranova, V., & Porter, D. W. (2013). Distribution and fibrotic response following inhalation exposure to multi-walled carbon nanotubes. Particle and Fibre Toxicology, 10, 33. https://doi.org/10.1186/1743-8977-10-33

Murphy, F. A., Schinwald, A., Poland, C. A., & Donaldson, K. (2012). The mechanism of pleural inflammation by long carbon nanotubes: interaction of long fibres with macrophages stimulates them to amplify pro-inflammatory responses in mesothelial cells. Particle and Fibre Toxicology, 9(1), 8. https://doi.org/10.1186/1743-8977-9-8

Murphy, F., Dekkers, S., Braakhuis, H., Ma-Hock, L., Johnston, H., Janer, G., di Cristo, L., Sabella, S., Jacobsen, N. R., Oomen, A. G., Haase, A., Fernandes, T., & Stone, V. (2021). An integrated approach to testing and assessment of high aspect ratio nanomaterials and its application for grouping based on a common mesothelioma hazard. NanoImpact, 100314. https://doi.org/https://doi.org/10.1016/j.impact.2021.100314

Osmond-McLeod, M. J., Poland, C. A., Murphy, F., Waddington, L., Morris, H., Hawkins, S. C., Clark, S., Aitken, R., McCall, M. J., & Donaldson, K. (2011). Durability and inflammogenic impact of carbon nanotubes compared with asbestos fibres. Particle and Fibre Toxicology, 8, 15. https://doi.org/10.1186/1743-8977-8-15

Palomäki, J., Sund, J., Vippola, M., Kinaret, P., Greco, D., Savolainen, K., Puustinen, A., & Alenius, H. (2015). A secretomics analysis reveals major differences in the macrophage responses towards different types of carbon nanotubes. Nanotoxicology, 9(6), 719–728. https://doi.org/10.3109/17435390.2014.969346

Palomäki, J., Välimäki, E., Sund, J., Vippola, M., Clausen, P. A., Jensen, K. A., Savolainen, K., Matikainen, S., & Alenius, H. (2011). Long, Needle-like Carbon Nanotubes and Asbestos Activate the NLRP3 Inflammasome through a Similar Mechanism. ACS Nano, 5(9), 6861–6870. https://doi.org/10.1021/nn200595c

Porter, D. W., Hubbs, A. F., Chen, B. T., McKinney, W., Mercer, R. R., Wolfarth, M. G., Battelli, L., Wu, N., Sriram, K., Leonard, S., Andrew, M., Willard, P., Tsuruoka, S., Endo, M., Tsukada, T., Munekane, F., Frazer, D. G., & Castranova, V. (2013). Acute pulmonary dose-responses to inhaled multi-walled carbon nanotubes. Nanotoxicology, 7(7), 1179–1194. https://doi.org/10.3109/17435390.2012.719649

Sakamoto, Y., Hojo, M., Kosugi, Y., Watanabe, K., Hirose, A., Inomata, A., Suzuki, T., & Nakae, D. (2018). Comparative study for carcinogenicity of 7 different multi-wall carbon nanotubes with different physicochemical characteristics by a single intraperitoneal injection in male Fischer 344 rats. The Journal of Toxicological Sciences, 43(10), 587–600. https://doi.org/10.2131/jts.43.587

Sanchez, V. C., Weston, P., Yan, A., Hurt, R. H., & Kane, A. B. (2011). A 3-dimensional in vitro model of epithelioid granulomas induced by high aspect ratio nanomaterials. Particle and Fibre Toxicology, 8, 17. https://doi.org/10.1186/1743-8977-8-17

Seidel, C., Zhernovkov, V., Cassidy, H., Kholodenko, B., Matallanas, D., Cosnier, F., & Gaté, L. (2021). Inhaled multi-walled carbon nanotubes differently modulate global gene and protein expression in rat lungs. Nanotoxicology, 15(2), 238–256. https://doi.org/10.1080/17435390.2020.1851418

Takagi, A., Hirose, A., Nishimura, T., Fukumori, N., Ogata, A., Ohashi, N., Kitajima, S., & Kanno, J. (2008). Induction of mesothelioma in p53+/&minus; mouse by intraperitoneal application of multi-wall carbon nanotube. The Journal of Toxicological Sciences, 33(1), 105–116. https://doi.org/10.2131/jts.33.105

Umeda, Y., Kasai, T., Saito, M., Kondo, H., Toya, T., Aiso, S., Okuda, H., Nishizawa, T., & Fukushima, S. (2013). Two-week Toxicity of Multi-walled Carbon Nanotubes by Whole-body Inhalation Exposure in Rats. Journal of Toxicologic Pathology, 26(2), 131–140. https://doi.org/10.1293/tox.26.131

Zhu, W., von dem Bussche, A., Yi, X., Qiu, Y., Wang, Z., Weston, P., Hurt, R. H., Kane, A. B., & Gao, H. (2016). Nanomechanical mechanism for lipid bilayer damage induced by carbon nanotubes confined in intracellular vesicles. Proceedings of the National Academy of Sciences, 113(44), 12374 LP – 12379. https://doi.org/10.1073/pnas.1605030113
