# Supplementary material for: Grouping MWCNTs based on their similar potential to cause pulmonary hazard after inhalation: a case-study
Source: Part Fibre Toxicol. 2022 Jul 20;19:50. doi: 10.1186/s12989-022-00487-6 (PMC9297605; doi:10.1186/s12989-022-00487-6)
Supplement: Supplementary file 4 — Additional file 4: Table S4: Summary of inhalation studies assessing the hazard of NRCWE006, NM-401, and NM-403 MWCNT. [file 12989_2022_487_MOESM4_ESM.docx]

Additional File 4

Table S4: Summary of inhalation studies assessing the hazard of NRCWE006, NM-401, and NM-403 MWCNT.

| **MWCNT** | **Species** | **Exposure time** | **Post exposure time** | **Aerosol concentration** | **Exposure mode** | **Key results: Inflammatory markers** | **Key results: Histopathology** |
| --- | --- | --- | --- | --- | --- | --- | --- |
| NM-401  (Gaté et al., 2019) | Rat | 4 weeks  (2 x 3 h/day, 5 days/week) | 3 days  30 days  90 days  180 days | 0.5 and 1.5mg/m^3^ | Nose-only | 20-fold increase in neutrophil influx at day 3, reduced to a 10-fold increase at days 30 (p < .0001) and 90 (p = .0002) and was still visible, though not significant, at 180 days post exposure. | Presence of macrophages containing black particles consistent with carbon nanotubes observed between 3 and 180 days post-exposure. By 180 days post exposure, a smaller number of positive macrophages were present in alveoli, and the number of macrophage aggregates associated with bronchioles was reduced. No significant difference in the amount or distribution of collagen in the lung was observed. |
| NM-403  (Gaté et al., 2019) | Rat | 4 weeks  (2 x 3 h/day, 5 days/week) | 3 days  30 days  90 days  180 days | 0.5 and 1.5mg/m^3^ | Nose-only | Increase in the number of neutrophils (between 15 and 20-fold) for both concentrations 3 days after the end of exposure compared to matched air-exposed controls (p < .0001 for both doses). Influx was still observed after 30 (p < .0001 for both doses) and 90 days (p < .0001 for the highest dose), but it decreased in a dose-dependentmanner. 180 days after the end of exposure, an increase in neutrophilic granulocytes was still significant for both doses of NM-403 (p < .0001). | At days 3, 30, 90 and 180, all NM-403-exposed rats showed accumulation of dark material in the cytoplasm of alveolar macrophages in the lungs which was not accompanied by any treatment-related inflammatory change or other alteration of the lung parenchyma. No exposure-related changes such as interstitial fibrosis were observed. |
| NM-401  (Seidel et al., 2021) | Rat | 4 weeks  (2 x 3 h/day, 5 days/week) | 3 days  30 days  90 days  180 days | 0.5 and 1.5mg/m^3^ | Nose-only | Proteome in the bronchoalveolar lavage fluid was analysed. Dose-dependent increase in the number of differentially expressed proteins measured. Number of differentially expressed proteins remained elevated over time. | Transcriptome in the whole lung was analysed. Dose-dependent increase in the number of differentially expressed genes identified which decreased over time. Eight genes (from the DEGs common across all times) commonly deregulated in the ‘fibrosis gene list’ from Snyder-Talkington et al. (2013) were identified. |
| NM-403  (Seidel et al., 2021) | Rat | 4 weeks  (2 x 3 h/day, 5 days/week) | 3 days  30 days  90 days  180 days | 0.5 and 1.5mg/m^3^ | Nose-only | NM-403 modulated fewer but more constant number of differentially expressed proteins compared to NM-401 at all post-exposure times. | NM-403 modulated fewer but more constant number of differentially expressed genes compared to NM-401 at all post-exposure times. |
| MWNT-7  (NRCWE006) (Umeda et al., 2013) | Rat | 2 weeks  (6 h/day, 5 days/week) | 4 weeks | 0.2, 1, 5mg/m^3^ | Whole body | Increase in the number of neutrophils in rats exposed to 1 and 5 mg/m^3^ at end of 2-week exposure.  After 4-week post exposure, number of neutrophils remained increased in 5mg/m^3^ group. | Granulomatous changes (aggregation of MWCNT-containing alveolar macrophages with some collagen fibre deposition) in the lung were observed in the rats exposed to 5mg/m^3^ MWCNT at the end of the 2 week.  The incidence of granulomatous changes had increased by 4-week postexposure period. |
| MWNT-7  (NRCWE006)  (Kasai et al., 2015) | Rat | 13 weeks  (6 h/day, 5 days/week) | - | 0.2, 1, 5mg/m^3^ | Whole body | Exposure concentration-related increases in the number of neutrophils and lymphocytes were observed in all MWCNT-exposed male and female rats. Percentages of bi- and multinucleated macrophages were significantly increased in all MWCNT-exposed male and female rats. | Granulomatous changes (increased collagen fibers, aggregation of MWCNT-phagocytosing alveolar macrophages and Langhans-like giant cells) ,were found in the alveolar region near the terminal bronchioles in rats exposed to 1 and 5 mg/m^3^ MWCNTs and focal fibrosis of the alveolar wall was observed in the alveolar ducts in rats exposed to 1 and 5 mg/m^3^ MWCNTs. |
| MWNT-7  (NRCWE006) (Kasai et al., 2016) | Rat | 104 weeks  (6 h/day, 5 days/week) | - | 0, 0.02, 0.2, and 2 mg/m^3^ | Whole body | The numbers of neutrophils, eosinophils, lymphocytes,  and macrophages were increased in the lavage fluid of males and females concentration-dependently and neutrophils, eosinophils, and lymphocytes were significantly elevated in 2 mg/m^3^ MWNT-7 exposed males and females. | Concentration-dependent toxic effects in the lung such as epithelial hyperplasia, granulomatous change, localized fibrosis were found in MWNT-7 treatment groups of both sexes (0.2 and 2mg/m^3^). Lung carcinomas, mainly bronchiolo-alveolar carcinoma, and combined carcinomas and adenomas were  significantly increased in rats exposed to 0.2 and 2 mg/m^3^ MWNT-7 compared to the clean air controls. |
| Mitsui-7  (NRCWE006) (Mercer et al., 2013) | Mouse | 3 weeks  (5 h/day, 4 days/week) | 1 day  14 days  84 days  168 days  336 days | 5mg/m^3^ | Whole body | Number of neutrophils increased rapidly (1 day post) after inhalation of MWCNTs and declined slowly with time post-exposure remaining significantly elevated at 168 days post exposure. At 336 days post-exposure neutrophil numbers still elevated above controls but not statistically different. | A trend toward increased fibrillar collagen within alveolar septa is apparent at 14 days post-exposure based on Sirius Red binding to fibrillar collagen. The increase in fibrillar collagen was significantly elevated  over control at 84, 168 and 336 days post-exposure and increases by 62, 56 and 70% above 1 day post-exposure, respectively. |

Gaté, L., Knudsen, K. B., Seidel, C., Berthing, T., Chézeau, L., Jacobsen, N. R., Valentino, S., Wallin, H., Bau, S., Wolff, H., Sébillaud, S., Lorcin, M., Grossmann, S., Viton, S., Nunge, H., Darne, C., Vogel, U., & Cosnier, F. (2019). Pulmonary toxicity of two different multi-walled carbon nanotubes in rat: Comparison between intratracheal instillation and inhalation exposure. *Toxicology and Applied Pharmacology*, *375*, 17–31. https://doi.org/https://doi.org/10.1016/j.taap.2019.05.001

Kasai, T., Gotoh, K., Nishizawa, T., Sasaki, T., Katagiri, T., Umeda, Y., Toya, T., & Fukushima, S. (2014). Development of a new multi-walled carbon nanotube (MWCNT) aerosol generation and exposure system and confirmation of suitability for conducting a single-exposure inhalation study of MWCNT in rats. *Nanotoxicology*, *8*(2), 169–178. https://doi.org/10.3109/17435390.2013.766277

Kasai, T., Umeda, Y., Ohnishi, M., Kondo, H., Takeuchi, T., Aiso, S., Nishizawa, T., Matsumoto, M., & Fukushima, S. (2015). Thirteen-week study of toxicity of fiber-like multi-walled carbon nanotubes with whole-body inhalation exposure in rats. *Nanotoxicology*, *9*(4), 413–422. https://doi.org/10.3109/17435390.2014.933903

Kasai, T., Umeda, Y., Ohnishi, M., Mine, T., Kondo, H., Takeuchi, T., Matsumoto, M., & Fukushima, S. (2016). Lung carcinogenicity of inhaled multi-walled carbon nanotube in rats. *Particle and Fibre Toxicology*, *13*(1), 53. https://doi.org/10.1186/s12989-016-0164-2

Mercer, R. R., Scabilloni, J. F., Hubbs, A. F., Battelli, L. A., McKinney, W., Friend, S., Wolfarth, M. G., Andrew, M., Castranova, V., & Porter, D. W. (2013). Distribution and fibrotic response following inhalation exposure to multi-walled carbon nanotubes. *Particle and Fibre Toxicology*, *10*, 33. https://doi.org/10.1186/1743-8977-10-33

Seidel, C., Zhernovkov, V., Cassidy, H., Kholodenko, B., Matallanas, D., Cosnier, F., & Gaté, L. (2021). Inhaled multi-walled carbon nanotubes differently modulate global gene and protein expression in rat lungs. *Nanotoxicology*, *15*(2), 238–256. https://doi.org/10.1080/17435390.2020.1851418

Umeda, Y., Kasai, T., Saito, M., Kondo, H., Toya, T., Aiso, S., Okuda, H., Nishizawa, T., & Fukushima, S. (2013). Two-week Toxicity of Multi-walled Carbon Nanotubes by Whole-body Inhalation Exposure in Rats. *Journal of Toxicologic Pathology*, *26*(2), 131–140. https://doi.org/10.1293/tox.26.131
