# Supplementary material for: Grouping MWCNTs based on their similar potential to cause pulmonary hazard after inhalation: a case-study
Source: Part Fibre Toxicol. 2022 Jul 20;19:50. doi: 10.1186/s12989-022-00487-6 (PMC9297605; doi:10.1186/s12989-022-00487-6)
Supplement: Supplementary file 5 — Additional file 5: Fig. S1: NRCWE006, NM-401, NRCWE040 dispersed by ultrasonication in 0.5% BSA/RPMI cell culture media prior to exposure to cells. NRCWE006, NM-401, NRCWE040 and THP-1 cells 24 hours after exposure. [file 12989_2022_487_MOESM5_ESM.docx]

Additional File 5


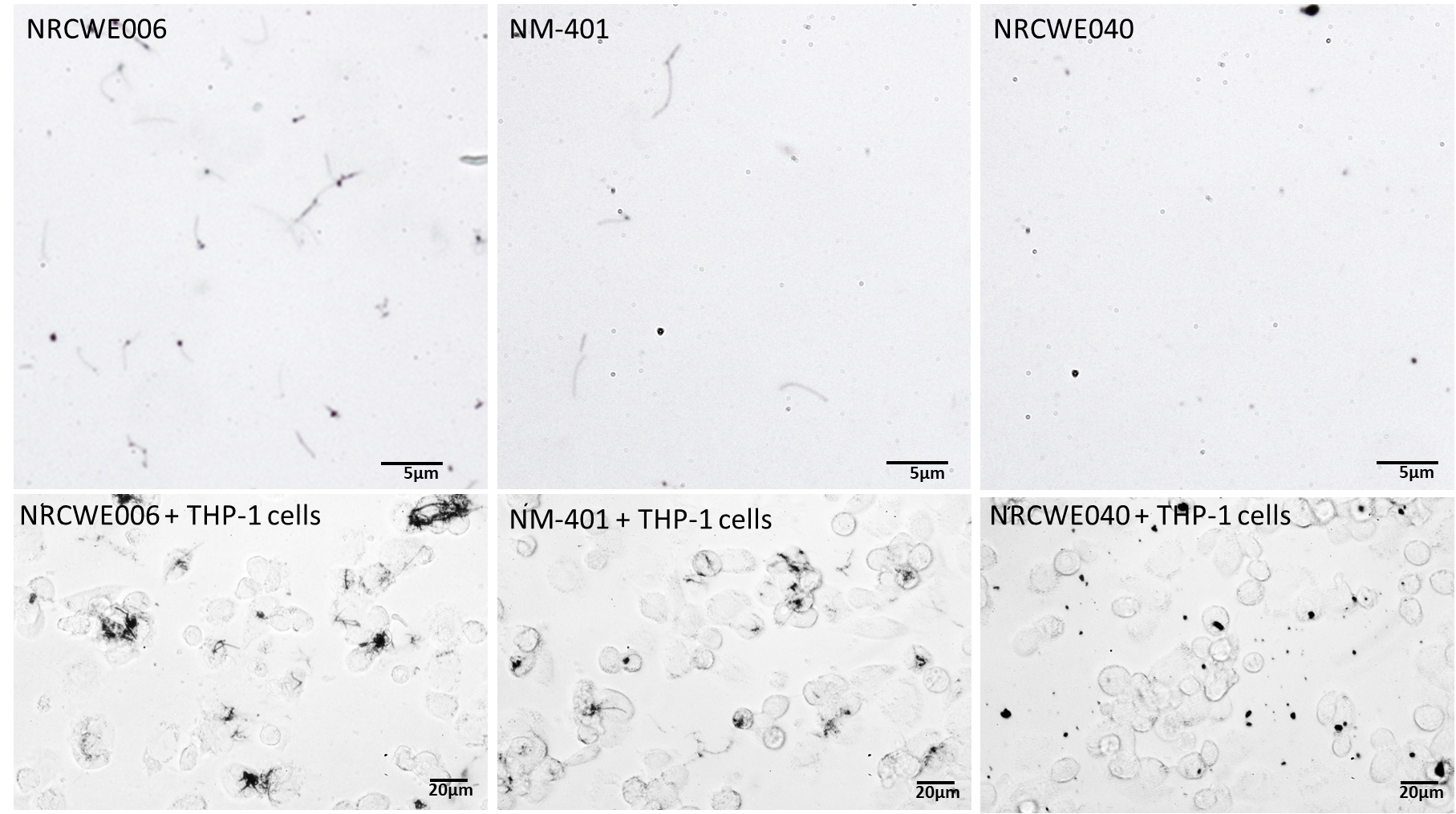


Figure S1: NRCWE006, NM-401, NRCWE040 dispersed by ultrasonication in 0.5% BSA/RPMI cell culture media prior to exposure to cells. NRCWE006, NM-401, NRCWE040 and THP-1 cells 24 hours after exposure.
