# Supplementary material for: Grouping MWCNTs based on their similar potential to cause pulmonary hazard after inhalation: a case-study
Source: Part Fibre Toxicol. 2022 Jul 20;19:50. doi: 10.1186/s12989-022-00487-6 (PMC9297605; doi:10.1186/s12989-022-00487-6)
Supplement: Supplementary file 6 — Additional file 6: Fig. S2: Scanning microscopy images of MWCNT panel adapted from (Jackson et al., 2015). Jackson, P., Kling, K., Jensen, K. A., Clausen, P. A., Madsen, A. M., Wallin, H., & Vogel, U. (2015). Characterization of genotoxic response to 15 multiwalled carbon nanotubes with variable physicochemical properties including surface functionalizations in the FE1-Muta(TM) mouse lung epithelial cell line. Environmental and Molecular Mutagenesis, 56(2), 183–203. [file 12989_2022_487_MOESM6_ESM.docx]

Additional File 6
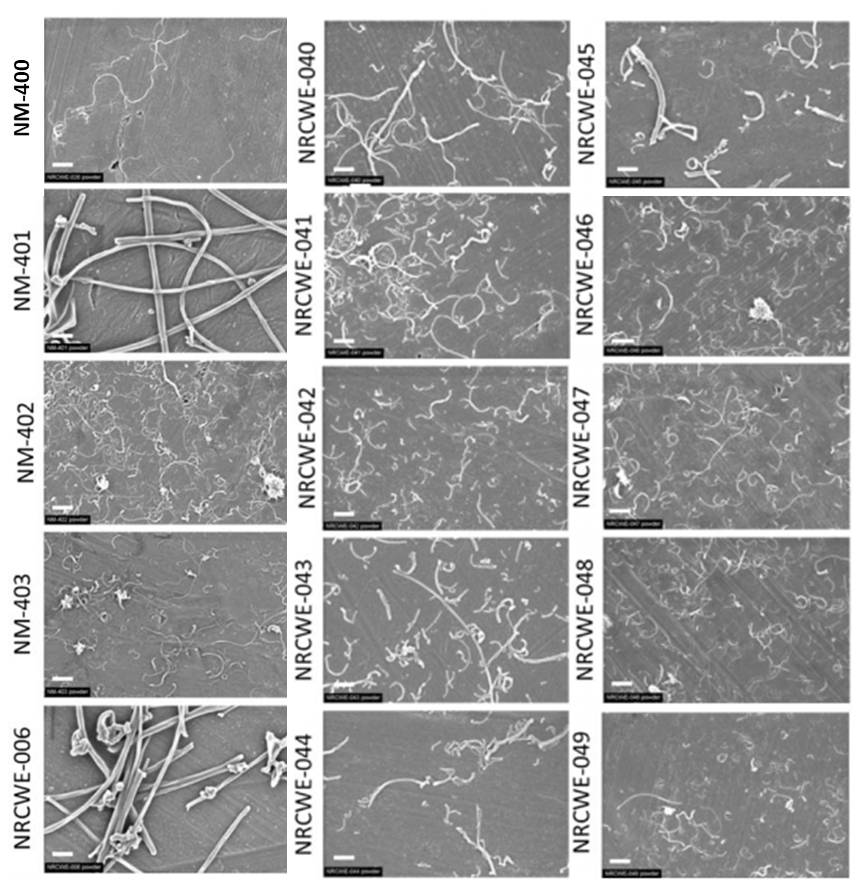


Figure S2: Scanning microscopy images of MWCNT panel adapted from (Jackson et al., 2015).

Jackson, P., Kling, K., Jensen, K. A., Clausen, P. A., Madsen, A. M., Wallin, H., & Vogel, U. (2015). Characterization of genotoxic response to 15 multiwalled carbon nanotubes with variable physicochemical properties including surface functionalizations in the FE1-Muta(TM) mouse lung epithelial cell line. Environmental and Molecular Mutagenesis, 56(2), 183–203. https://doi.org/https://doi.org/10.1002/em.21922
