# Supplementary material for: Grouping MWCNTs based on their similar potential to cause pulmonary hazard after inhalation: a case-study
Source: Part Fibre Toxicol. 2022 Jul 20;19:50. doi: 10.1186/s12989-022-00487-6 (PMC9297605; doi:10.1186/s12989-022-00487-6)
Supplement: Supplementary file 7 — Additional file 7: Table S5: Fold change over no particle control, DCFH Fluorescent arbitrary units. Table S6: IL-1β release from THP-1 cells exposure to MWCNT for 24 hours measured in supernantant by ELISA (R&D systems Duo Set). Exposure co ncentration range 1.56-100µg/ml. [file 12989_2022_487_MOESM7_ESM.docx]

Additional File 7

Experimental raw data

DCFH_2_-DA raw data

Table S5: Fold change over no particle control, DCFH Fluorescent arbitrary units.

| ug/ml | NRCWE006 | NM-401 | NRCEW040 | NPCB |
| --- | --- | --- | --- | --- |
| 1.6 | 0.852304 | 0.704719 | 1.055342 | 1.472788 |
| 3.1 | 0.839945 | 0.717572 | 1.136566 | 1.775058 |
| 6.25 | 0.855834 | 0.715019 | 1.455708 | 2.049662 |
| 12.5 | 0.858181 | 0.717079 | 2.035223 | 2.545914 |

IL-1β release

ELISA raw data

Table S6: IL-1β release from THP-1 cells exposure to MWCNT for 24 hours measured in supernantant by ELISA (R&D systems Duo Set). Exposure concentration range 1.56-100µg/ml.

|  | NRCWE006 | NM-401 |
| --- | --- | --- |
| 0 | 17.92014 | 17.92014 |
| 1.56 | 45.67048 | 17.91154 |
| 3.125 | 70.78596 | 36.46619 |
| 6.25 | 124.7662 | 72.19324 |
| 12.5 | 215.919 | 128.3419 |
| 25 | 306.1034 | 263.7737 |
| 50 | 333.6344 | 334.6191 |
| 100 | 356.763 | 378.7641 |
