# Supplementary material for: Grouping MWCNTs based on their similar potential to cause pulmonary hazard after inhalation: a case-study
Source: Part Fibre Toxicol. 2022 Jul 20;19:50. doi: 10.1186/s12989-022-00487-6 (PMC9297605; doi:10.1186/s12989-022-00487-6)
Supplement: Supplementary file 8 — Additional file 8: Table S6: Residual metal content for MWCNT case study panel. Data adapted from Jackson et al 2015. [file 12989_2022_487_MOESM8_ESM.docx]

Additional File 8

Table S5: Residual metal content for MWCNT case study panel. Data adapted from Jackson et al 2015

|  | Al_2_O_3_ | BaO | CaO | Cl | CoO | Cr_2_O_3_ | CuO | Fe_2_O_3_ | K_2_O | La_2_O_3_ | MgO | MnO | NiO | P_2_O_5_ | SiO_2_ | SO_3_ | ZnO |
| --- | --- | --- | --- | --- | --- | --- | --- | --- | --- | --- | --- | --- | --- | --- | --- | --- | --- |
| NM-400 | 4.59 |  | 0.01 | 0.01 | 0.11 | 0.002 | 0.0007 | 0.29 |  |  |  |  | 0.0008 |  |  |  | 0.002 |
| NM-401 |  |  | 0.03 |  |  |  | 0.0002 | 0.05 |  |  | 0.01 |  |  | 0.14 | 0.009 | 0.08 | 0.001 |
| NM-402 | 2.43 |  | 0.03 |  |  |  | 0.0005 | 1.31 |  |  | 0.001 |  | 0.0011 | 0.16 | 0.01 |  | 0.001 |
| NM-403 | 0.24 |  | 0.03 |  | 0.12 |  | 0.0003 | 0.002 |  |  | 0.19 | 0.16 | 0.0018 | 0.14 |  |  | 0.001 |
| NRCWE-006 |  |  | 0.03 |  |  |  | 0.0003 | 0.08 |  |  | 0.01 |  |  | 0.14 | 0.006 | 0.08 | 0.001 |
| NRCWE-040 |  |  | 0.05 | 0.05 | 0.001 | 0.02 | 0.0013 | 0.2 |  | 0.32 | 0.01 | 0.002 | 0.56 | 0.15 | 0.02 | 0.08 | 0.001 |
| NRCWE-041 |  |  | 0.13 | 0.02 | 0.001 | 0.02 |  | 0.13 | 0.003 | 0.03 | 0.02 | 0.001 | 0.31 | 0.15 | 0.02 | 0.01 | 0.001 |
| NRCWE-042 | 0.01 |  | 0.25 | 0.02 | 0 | 0.008 | 0.0007 | 0.08 | 0.005 | 0.02 | 0.03 | 0.001 | 0.21 | 0.14 | 0.01 | 0.01 | 0.001 |
| NRCWE-043 |  |  | 0.04 |  | 0.001 | 0.002 |  | 0.008 | 0.001 | 0.02 | 0.01 |  | 1.2 | 0.15 | 0.006 | 0.04 | 0.001 |
| NRCWE-044 |  |  | 0.08 | 0.01 | 0.002 |  | 0.0024 | 0.004 | 0.003 | 0.01 | 0.02 |  | 1.04 | 0.14 | 0.01 | 0.03 | 0.001 |
| NRCWE-045 | 0.52 | 0.006 | 0.08 | 0.02 | 0.25 | 0.02 | 0.0038 | 1.17 | 0.003 | 0.01 | 0.02 | 0.002 | 1.34 | 0.16 | 0.02 | 0.06 | 0.001 |
| NRCWE-046 | 0.29 |  | 0.03 | 0.01 | 0.25 | 0.002 | 0.0015 | 0.008 |  |  | 0.22 | 0.3 | 0.0045 | 0.14 | 0.007 |  | 0.001 |
| NRCWE-047 | 0.27 |  | 0.03 | 0.02 | 0.25 | 0.001 | 0.0006 | 0.007 |  |  | 0.22 | 0.3 | 0.0043 | 0.15 | 0.02 | 0.01 | 0.001 |
| NRCWE-048 | 0.26 |  | 0.02 |  | 0.24 | 0.001 |  | 0.007 |  |  | 0.19 | 0.28 | 0.0037 | 0.14 | 0.007 |  | 0.001 |
| NRCWE-049 | 0.26 |  | 0.03 |  | 0.25 | 0.001 | 0.0004 | 0.004 |  |  | 0.19 | 0.29 | 0.0038 | 0.15 | 0.008 | 0.01 | 0.001 |
