# Supplementary material for: Grouping MWCNTs based on their similar potential to cause pulmonary hazard after inhalation: a case-study
Source: Part Fibre Toxicol. 2022 Jul 20;19:50. doi: 10.1186/s12989-022-00487-6 (PMC9297605; doi:10.1186/s12989-022-00487-6)
Supplement: Supplementary file 9 — Additional file 9: Fig. S3: Transmission Electron microscope images of MWCNT panel. Scale bar = 100µm. MWCNT were dispersed in 70% ethanol and dispersed by ultrasonication prior to mounting on TEM grids. [file 12989_2022_487_MOESM9_ESM.docx]

Additional File 9


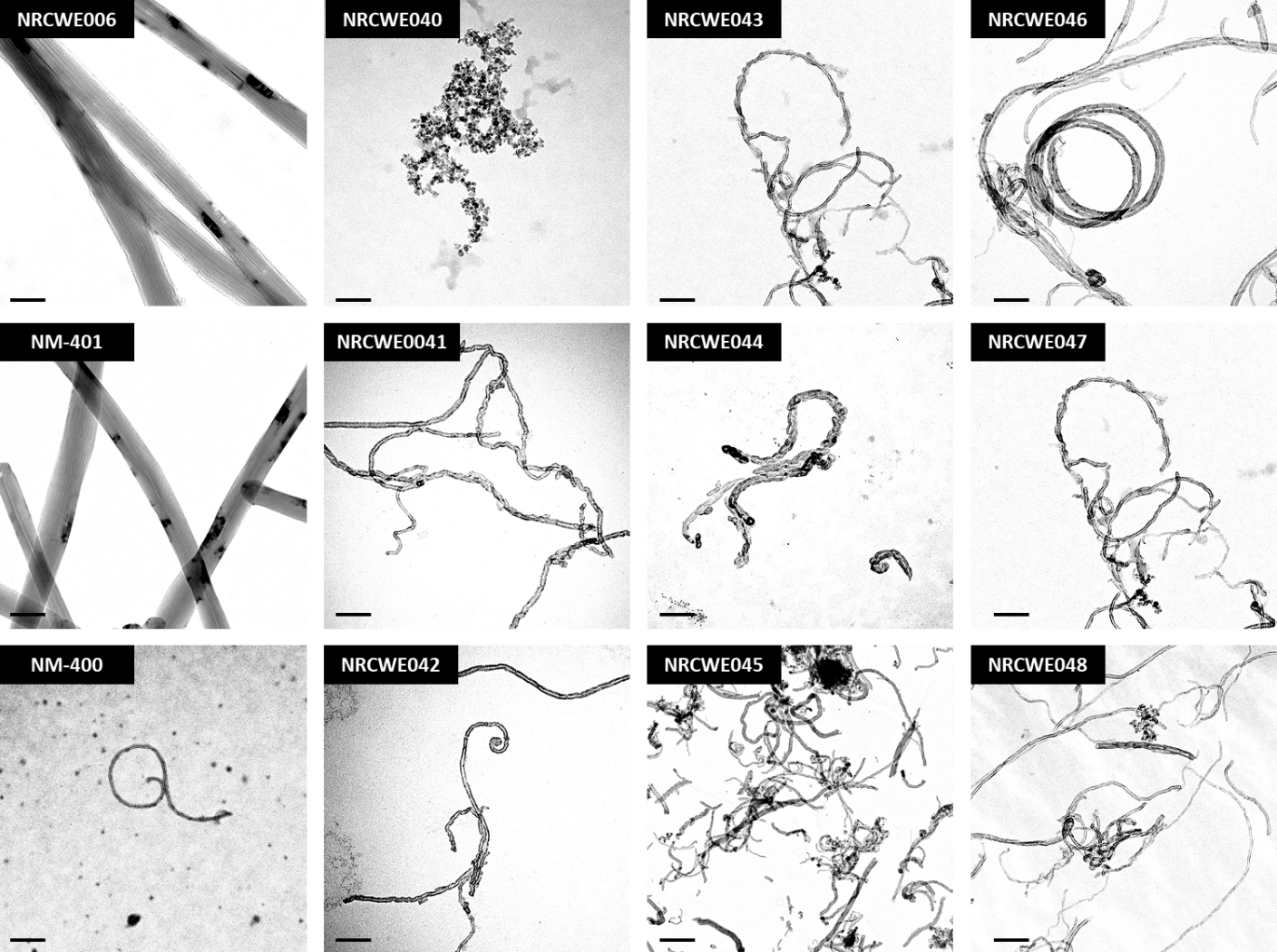


Figure S3: Transmission Electron microscope images of MWCNT panel. Scale bar = 100µm. MWCNT were dispersed in 70% ethanol and dispersed by ultrasonication prior to mounting on TEM grids.
