# Supplementary material for: Grouping MWCNTs based on their similar potential to cause pulmonary hazard after inhalation: a case-study
Source: Part Fibre Toxicol. 2022 Jul 20;19:50. doi: 10.1186/s12989-022-00487-6 (PMC9297605; doi:10.1186/s12989-022-00487-6)
Supplement: Supplementary file 10 — Additional file10: Fig. S4: Light microscopy images of THP-1 cells exposed to MWCNT panel (10µg/ml) for 6 hours. Scale bar = 20µm. [file 12989_2022_487_MOESM10_ESM.docx]

Additional File 10


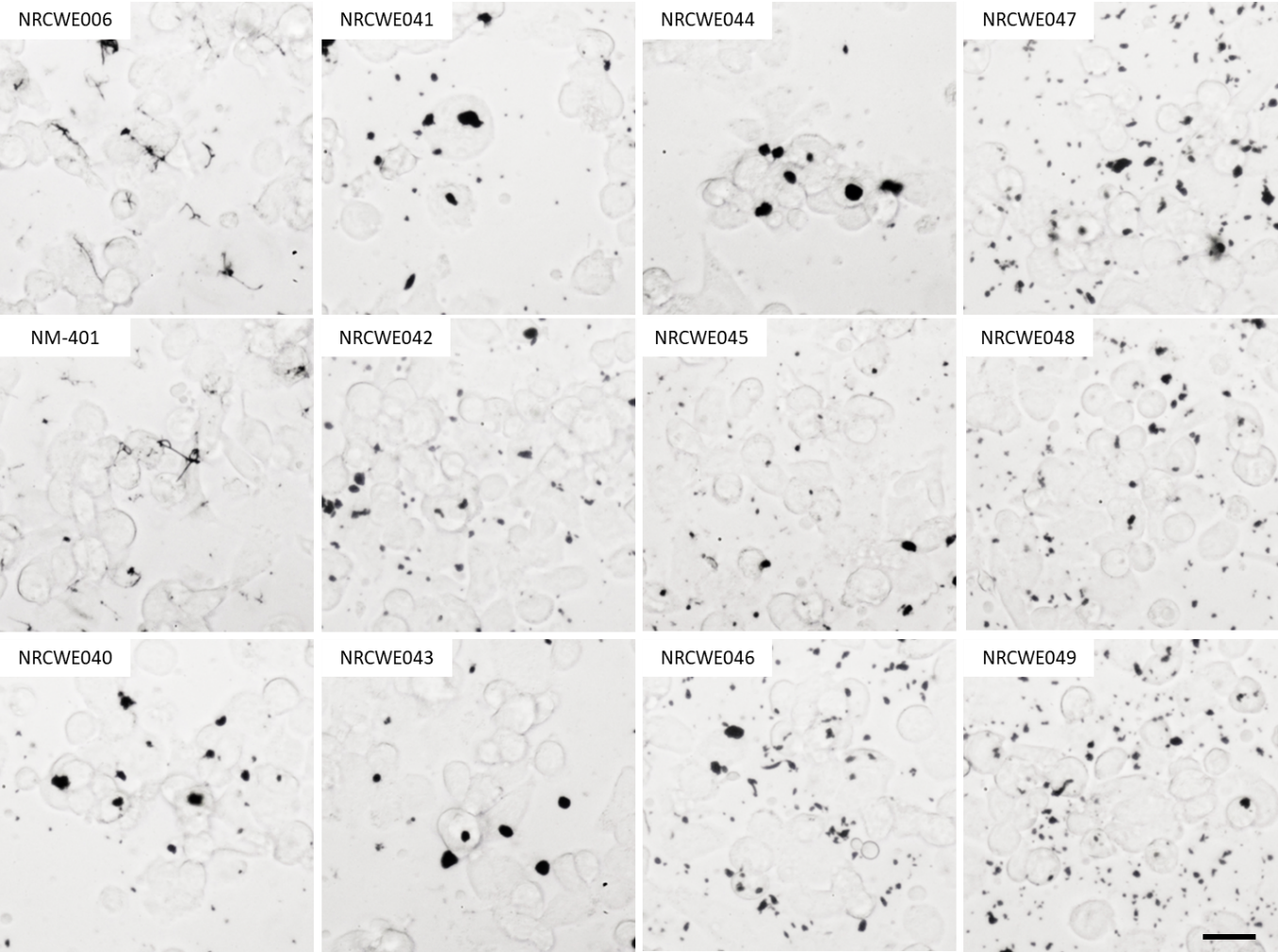


Figure S4: Light microscopy images of THP-1 cells exposed to MWCNT panel (10µg/ml) for 6 hours. Scale bar = 20µm.
